# Supplementary material for: A self-efficacy education programme on foot self-care behaviour among older patients with diabetes in a public long-term care institution, Malaysia: a Quasi-experimental Pilot Study
Source: BMJ Open. 2017 Jun 8;7(6):e014393. doi: 10.1136/bmjopen-2016-014393 (PMC5623401; doi:10.1136/bmjopen-2016-014393)
Supplement: Author's manuscript [file bmjopen-2016-014393.draft_revisions.pdf]

A Self-Efficacy Education Program on Foot Self-Care Behaviour among Older Diabetics in a Public Long-Term Care Institution, Malaysia: A Pilot Study

Journal:	*BMJ Open*
Manuscript ID	bmjopen-2016-014393
Article Type:	Research
Date Submitted by the Author:	21-Sep-2016
Complete List of Authors:	Sharoni, Siti Khuzaimah Ahmad; Univ Teknol MARA, Nursing Department; University Putra Malaysia, Community Health Department<br>Abdul Rahman, Hejar; University Putra Malaysia, Community Health Department, Faculty of Medicine and Health Sciences<br>Minhat, Halimatus Sakdiah; University Putra Malaysia, Community Health Department, Faculty of Medicine and Health Sciences<br>Shariff Ghazali, Sazlina; University Putra Malaysia, Department of Family Medicine, Faculty of Medicine and Health Sciences<br>Azman Ong, Mohd Hanafi; Universiti Teknologi MARA, Department of Statistics, Faculty of Computer Science and Mathematics, UiTM Segamat Campus
**Primary Subject Heading**:	Diabetes and endocrinology
Secondary Subject Heading:	Geriatric medicine, Nursing, Public health
Keywords:	Diabetic foot < DIABETES & ENDOCRINOLOGY, PUBLIC HEALTH, GERIATRIC MEDICINE

A Self-Efficacy Education Program on Foot Self-Care Behaviour among Older Diabetics in a Public Long-Term Care Institution, Malaysia: A Pilot Study

International naming convention:

Sharoni, S.K.A, Rahman, H.A, Minhat, H. S., Ghazali, S.S, & Ong, M.H.A

Authors' addresses

Siti Khuzaimah Ahmad Sharoni[1,2], Hejar Abdul Rahman[2*], Halimatus Sakdiah Minhat[2], Sazlina Shariff Ghazali[3], Mohd Hanafi Azman Ong[4]

[1]Department of Nursing, Faculty of Health Sciences, Universiti Teknologi MARA, Puncak Alam Campus, 42300 Puncak Alam, Selangor, Malaysia

[2]Department of Community Health, Faculty of Medicine and Health Sciences, Universiti Putra Malaysia, 43400 UPM Serdang, Selangor, Malaysia

[3]Department of Family Medicine, Faculty of Medicine and Health Sciences, Universiti Putra Malaysia 43400 UPM Serdang, Selangor, Malaysia

[4]Department of Statistics, Faculty of Computer Science and Mathematics, Universiti Teknologi MARA, Segamat Campus, KM 12, Jalan Muar, 85000, Segamat, Johor, Malaysia.

Authors' information:

1) Siti Khuzaimah Ahmad Sharoni (MscN, RN)

Lecturer, Department of Nursing, Faculty of Health Sciences, Universiti Teknologi MARA, Puncak Alam Campus, 42300 Puncak Alam, Selangor, Malaysia

PhD student, Department of Community Health, Faculty of Medicine and Health Sciences, Universiti Putra Malaysia, 43400 UPM Serdang, Selangor, Malaysia

Email: sitik123@yahoo.com ; sitik123@salam.uitm.edu.my

Telephone: +603-3258 4349; Fax: +603-3258 4599

2) Associate Professor Dr Hejar Abdul Rahman (M. Com. Health) ***Corresponding author**

Associate Professor, Department of Community Health, Faculty of Medicine and Health Sciences, Universiti Putra Malaysia, 43400 UPM Serdang, Selangor, Malaysia

Email: hejar@upm.edu.my

Telephone: +603-8947 2413; Fax: +603-89450151

For peer review only

For peer review only - http://bmjopen.bmj.com/site/about/guidelines.xhtml

3) Associate Professor Dr Halimatus Sakdiah Minhat (DrPh)

Associate Professor, Department of Community Health, Faculty of Medicine and Health Sciences, Universiti Putra Malaysia, 43400 UPM Serdang, Selangor, Malaysia

Email: halimatus@upm.edu.my

Telephone: +603-8947 2417; Fax: +603-89450151

4) Associate Professor Dr Sazlina Shariff Ghazali (PhD, M. Med (Fam. Med),

Associate Professor, [3]Department of Family Medicine, Faculty of Medicine and Health Sciences, Universiti Putra Malaysia 43400 UPM Serdang, Selangor, Malaysia

Email: sazlina@upm.edu.my

Telephone: +603-8947 2532; Fax: +603-89472328

5) Mohd Hanafi Azman Ong (Msc. Applied Statistics)

Lecturer, Department of Statistics, Faculty of Computer Science and Mathematics, Universiti Teknologi MARA, Segamat Campus, KM 12, Jalan Muar, 85000, Segamat, Johor, Malaysia.

Email: napieong@yahoo.com

Telephone: +607-935 2000; Fax: +607-935 2277

Keywords: Diabetes, foot, long-term care, self-care behaviour, self-efficacy

Statistical summary of the manuscript:

Manuscript content: 4360 words

Abstract: 257 words

No of table/ figure: 5 tables, 1 Figure

No of references: 47 References

Data sharing statement: Our study protocol can be accessed freely available via https://www.anzctr.org.au/Trial/Registration/TrialReview.aspx?id=369488

For peer review only

ABSTRACT

Objective: A self-efficacy education program was piloted to access the feasibility, acceptability and potential impact of the self-efficacy education program on improving the foot self-care behaviour among older diabetics in a public long-term care institution.

Method: A pre- and post- quasi-experimental study was conducted in a public long-term care institution in Selangor, Malaysia. Diabetes patients aged 60 years and above that fulfilled the selection criteria were invited to participate in this program. Four self-efficacy information sources; mastery experience, modelling, psychological states, and verbal persuasion were translated into program interventions. The program consisted of 20 minutes of seminar presentation, routine weekly visits and follow-up for problem-solving and support at week-4 and week-12. The primary outcome was foot self-care behaviour. Foot self-efficacy (efficacy-expectation), foot care outcome expectation, knowledge on foot care, quality of life, fasting blood glucose and foot condition were secondary outcomes. Data were analysed with descriptive and inferential statistics (McNemar test and Wilcoxon signed-rank test) using Statistical Package for the Social Sciences version 20.0.

Results: Fifty-two people were recruited but later only 31 met the inclusion criteria and were analysed from baseline to 12 weeks of evaluation. The acceptability rate was moderately high. Post-intervention, foot self-care behaviour ($p<0.001$), foot self-efficacy (efficacy-expectation), ($p<0.001$), foot care outcome expectation ($p<0.001$), knowledge on foot care ($p<0.001$), quality of life (physical symptoms) ($p=0.003$), fasting blood glucose ($p=0.010$), foot hygiene ($p=0.030$) and anhydrosis ($p=0.020$) showed significant improvement.

Conclusion: Findings from this pilot study would facilitate planning of a larger study among older diabetes population living in long-term care institutions.

Trial registration number: ACTRN12616000210471

For peer review only

For peer review only - http://bmjopen.bmj.com/site/about/guidelines.xhtml

ARTICLE SUMMARY

Strengths and limitations of this study

- This is the first intervention program addressing foot self-care behaviour based on self-efficacy concept among older diabetics living in a public long-term care institution in Malaysia.
- The self-efficacy education program was feasible, acceptable and successful in assisting older diabetics to improve the foot self-care behaviour and other health outcomes.
- The study highlights the need to conduct a similar intervention among older diabetes living in long-term care institutions all over Malaysia.
- The sample is relatively small, and the intervention employed a non-randomised controlled trials since there was no control group there was at high potential biases.
- Self-reported in the outcome measurements may lead to recall and reporting bias. Other measurements such as data from medical record, biometric, or laboratory investigations would provide additional reliable and valid results.

A funding statement:

This work was supported by Universiti Putra Malaysia (Puta Grant), grant number 9463500

For peer review only

INTRODUCTION

Diabetes is a common chronic disease affecting older people, and it is becoming a global health concern. The International Diabetes Federation reported there are more than 134.6 million older diabetics worldwide, and the number is expected to increase to 252.8 million by 2035.[1] The prevalence of diabetes is expected to increase exponentially for the next 20 years in developing countries.[2] By 2030, there would be more than 82 million older diabetics in developing countries.[3]

In Malaysia, the National Health Morbidity Survey reported 15.2% (2.6 million) of people were diagnosed with diabetes.[4] The prevalence of diabetes among people aged 55 years and above was 45%.[5] Microvascular and severe late diabetic complications were reported at 75% and 25.4%, respectively.[6] The National Diabetes Registry reported the prevalence of neuropathy, diabetic foot ulcer and amputation were 70%, 11.1% and 11.0%, respectively. These complications due to diabetes have a significant impact especially for older populations.[5]

In Malaysia, the Ministry of Women, Family and Community Development abbreviated as KPWKM, manages the public long-term care institutions. It was reported that 9% of the residents living in public long-term care institutions in Peninsular Malaysia have diabetes.[7] Older diabetics who stayed in the institutions receive treatment from the public health clinics under the Ministry of Health (MOH). However, little is known about diabetes management among older diabetics living in long-term care facilities.

Diabetes education is effective in improving foot self-care behaviour and preventing diabetic foot complications.[8] Self-care behaviour is the ability, knowledge, skills and confidence to make daily decisions.[9] Foot self-care behaviour is essential as this can improve health outcomes. However, foot self-care behaviour awareness among Malaysians with diabetes is relatively low. The National Orthopaedic Registry Malaysia reported approximately 17.6% of diabetics attended diabetic foot clinics, 22.8% keep on diabetes booklet, 23.2% apply moisturising lotion on their feet, 26.5% wear appropriate footwear, and 27.3% have received formal education in foot care.[10]

For peer review only - http://bmjopen.bmj.com/site/about/guidelines.xhtml

Self-efficacy is defined as confidence in one's ability to perform a particular behaviour and is expected to influence the likelihood of the behavioural occurrence.[11 12] The Self-efficacy Theory refers to the individuals' belief, feelings and their motivation. The two essential components of this theory are the expectation of the individual's ability (self-efficacy or efficacy-expectation) and determination to practise specific behaviour (outcome expectations).[13] Previous intervention studies showed significant improvement in foot self-care behaviour after implementing self-efficacy education strategies.[14-16] To date, few published intervention studies related to self-efficacy education programs focused on foot self-care behaviour among older diabetics living in a public long-term care institution in Malaysia.

AIM

The objective of this study was to access the feasibility, acceptability and potential impact of the self-efficacy education program on improving the foot self-care behaviour among older diabetics in a public long-term care institution. The primary outcome of the study was foot self-care behaviour. Foot care self-efficacy (efficacy-expectation), foot care outcome expectation, knowledge on foot care, quality of life, fasting blood glucose (FBG) and foot condition were the secondary outcomes.

For peer review only

METHOD

Design and sample

This is a pre- and post-quasi-experimental study conducted in a public long-term care institution located in Selangor, Malaysia. During the period of data collection (January 2016), there was a total of 191 residents of whom 52 have diabetes. Older diabetics aged ≥60 years, Malaysian, able to communicate in Malay and independent in the activity of daily livings (ADLs) were invited to participate in this study. Older diabetics with cognitive impairment, psychosis, severe depression or blind, mute and deaf were excluded from this study. The principal researcher conducted a screening process prior to sample selection.

Elements of self-efficacy in the education program

This study incorporated the self-efficacy construct to develop the education program (Bandura, 1977). Self-efficacy in a person can be increased through four components: 1) performance accomplishments, 2) vicarious experience, 3) verbal persuasion, and 4) physiological information. Self-efficacy in diabetes management includes building new goals, starts with small steps, identify the specific needs, give positive feedback and encouragement, and skills improvement and problem solving for respondents who are in difficult situations.[17]

In this study, self-efficacy constructs were integrated with the respondents through knowledge transfer (seminar presentation and pamphlet) and self-efficacy (self-confidence) enhancement activities. To enhance the respondents' self-efficacy, they were encouraged to develop their targets, work in small, realistic steps, be more focused, and have the confidence to perform the desired behaviour. A pamphlet (symbolic modelling) was provided for self-guidance. During follow-up meetings, the respondents who were reported as not being able to perform the foot self-care behaviour received specific guidance and encouragement by the principal researcher to participate in the recommended lifestyle adjustments. A sharing session was conducted among the respondents who have difficulty to perform the behaviour effectively, the researcher and the local nurse. Those respondents who managed to carry out the behaviour effectively were appointed as a mentor (live modelling) to other respondents who were not able to do so. The respondents received weekly visits by the local nurse for physical and emotional support.

For peer review only

Intervention module

Knowledge transfer was conducted during the health education program. The respondent received the information from power point presentation (PPT) (oral and visual information) and pamphlet (written and visual information). The program was conducted in the meeting room, consisted of 10-11 respondents/ group/ session, and was facilitated by the researcher. The education activities include a 20 minutes PPT seminar aided with a pamphlet. The topic of this intervention program was “Foot self-care for older diabetics”. The content and design of the PPT and pamphlet were adapted from standard diabetes organisations.[1 18 19] The content of the PPT, and the pamphlet highlighted the risk factors of diabetes foot complications, foot self-examination, daily foot hygiene and cleanliness, foot protection and prevention of foot-related complications.

The foot care package consisted of the pamphlet of foot self-care, a nail clipper, a water-based lotion and a small towel, and was given to the respondents after the seminar.

A checklist reminder was developed for the local nurse in charge of the clinic at the institution. The nurse also received instruction from the researcher to remind, give support and guidance to the respondents to perform the foot self-care behaviour daily. The respondents were advised to ask for guidance from the local nurse or their colleagues. The nurse is required to sign after visiting the respondents.

Variables and measurement

The feasibility of this study was measured by the respondents’ recruitment, attendance, attrition and compliance rates.[20] The acceptability profile was evaluated after completing the 12-week program with the modified version of the Abbreviated Acceptability Rating Profile.[21] The scale consisted of eight items used to assess respondents’ acceptability of the self-efficacy education program. There was a 5 point Likert scale [strongly disagree (1), disagree (2), neither disagree or agree (3), agree (4) and strongly agree (5)]. The score ranges from 8 to 40; higher score indicated that the respondents’ have good acceptability towards the program delivered.

For peer review only - http://bmjopen.bmj.com/site/about/guidelines.xhtml

A questionnaire was used to evaluate the impact of the intervention to the outcome measures. Demographic data consisted of age, gender, ethnicity, education level, marital status, having children, and duration of stay in the institution. FBG, diabetes duration, treatment of diabetes, co-morbidity, previous diabetes education received, current smoking status and current history of hospitalisation related to diabetes were assessed for clinical characteristics.

The primary outcome of this study was foot self-care behaviour measured using the modified version of Diabetes Foot Self-Care Behaviour Scale (DFSBS).[22] The original DFSBS has good validity and reliability (Cronbach α = 0.73), and test-retest reliability was 0.92.[22] The foot self-care behaviour consisted of two parts. In part 1, seven items were asked about how many days respondents performed the foot self-care behaviour for the past seven days (one week). Part 2 (nine items) was about the frequency in which respondents performed a certain foot self-care behaviour. The responses were rated as 5 point Likert scale [never/ 0 day per week (1), rarely/ 1-2days per week (2), sometimes/ 3-4 days per week (3), often/ 5-6 days per week (4) and always/ 7 days per week (5)].[22] The score ranges from 16 to 80; higher score indicates good foot self-care behaviour.

For foot care self-efficacy (efficacy-expectation), the modified version of the Foot Care Confidence Scale (FCCS) was used.[23] The reliability test of the original FCCS was high (Cronbach α = 0.92).[23] The foot care self-efficacy (efficacy-expectation), consisted of 10 items of 5 point Likert scale [strongly not confident (1), not confident (2), moderate confident (3), confident (4) and strongly confident (5)]. The score ranges from 10 to 50; higher score indicated high confidence to perform the foot self-care behaviour.

Foot care outcome expectation was developed based from previous literature.[12 23 24 25] The scale consisted of six items of 5 point Likert scale [strongly disagree (1), disagree (2), neither disagree or agree (3), agree (4) and strongly agree (5)]. The score ranges from 6 to 30; higher score indicated that the respondents had high confidence the foot self-care behaviour that he/she performed will have a good effect.

For peer review only - http://bmjopen.bmj.com/site/about/guidelines.xhtml

Knowledge of foot care was developed based on previous literature.[26-28] The instrument assessed the respondents' knowledge about risk factors, common diabetic foot complications and foot care. The scale consisted of 11 items with three possible answers (true, false and don't know). One mark was given for each correct answer. The score ranges from 0 to 11. A higher score indicated a good level of knowledge.

The modified version of the Neuropathy and Foot Ulcer Specific Quality of Life (FS-QoL) was used to assess the quality of life of the respondents.[29] The original instrument showed a good reliability (Cronbach $\alpha = 0.86 - 0.95$). The instrument was divided into physical symptoms (13 items) and psychosocial functioning (12 items). This is an ordinal scale divided into two sections. First, the respondents need to respond to the foot problems affecting their well-being [always (3), sometimes (2), never (1)] and psychosocial functioning [every time (3), seldom (2), none (1) or agree (3), neither agree or disagree (2) and disagree (3)]. In the second section the respondents were asked about whether the foot problems bother them [none (1), some (2), or very (3)]. The total score was calculated by multiplying the score between section one and section two. The score ranges for physical symptoms were from 13 to 117 and for psychosocial functioning was from 12 to 108. The lower score indicated good quality of life.

Foot condition was developed from previous literature.[30 31] The instrument assessed the overall hygiene, nail conditions, common skin conditions, other conditions and infections and complication. If respondents had a foot condition, it was rated as "1 point" for each component (left or right).

For peer review only - http://bmjopen.bmj.com/site/about/guidelines.xhtml

Validity and reliability

The intervention module and the instruments were assessed for face and content validity. This was to ensure that the materials were appropriate for the current local practice, cultural equivalent, and appropriate for the population and study location. Content Validity Ratio (CVR) of the instruments were assessed by six experts; one endocrinologist, two public health specialist, one family medicine specialist, one diabetic nurse educator and one older diabetic.

CVR = (2ng / N) – 1, was used for validity conformity.[32] Where ng is the number of panel experts, who thinks the item is good, and N is the total number of panel experts. In this approach, six panel experts were asked to indicate whether or not a measurement item in a set of items is "essential" or "useful but not essential" or "not necessary" to the operationalisation of a theoretical construct.[33] "Essential" items or assessment tasks are ones that best represent the goal and are desired. The value lies between -1.00 to +1.00, where a CVR = 0.00 means that 50% of the panel experts in the panel size of N believe that the portfolio task is essential thereby valid (Johnston & Wilkinson, 2009).[33] In this study, the items were excluded for CVR < 0.00.

Forward and back translation from English to Malay and back to English translation was performed by the two bi-language translators certified from the Institute of Language and Literature Malaysia. The second draft of the questionnaire was weighted carefully before data collection procedures.

The results of internal consistency or reliability tests were acceptable for foot self-care behaviour (Cronbach α = 0.68), foot care self-efficacy (efficacy-expectation) (Cronbach α = 0.91), foot care outcome expectation (Cronbach α = 0.88), knowledge on foot care (Cronbach α = 0.86) and quality of life (physical symptoms, Cronbach α = 0.68) (psychosocial functioning, Cronbach α = 0.68).

The data collection procedures were conducted by the principal researcher (registered nurse), a local nurse (community nurse who in charge the clinic) and a research enumerator (registered nurse). The research enumerator received two hours training with a manual file. The local nurse received a 30-minute briefing by the principal researcher regarding the visiting procedures and checklist usage.

For peer review only - http://bmjopen.bmj.com/site/about/guidelines.xhtml

Data collection procedures

The data collection process consisted of four visits. During the first visit, the activities included screening session of all older diabetics for inclusion and exclusion criteria, consent taking procedures and baseline assessment. The respondents received information about the study's objectives, procedures, benefits and potential harms by the main researcher. Data collection was conducted by a trained research enumerator from baseline to week-12.

The second visit was a 30-minute seminar presentation delivered by the principal researcher in a meeting room at the institution. The respondents were divided into three groups: group A (10 respondents), group B (10 respondents) and group C (11 respondents). The presentation was delivered three times (one group/ session). At the end of the seminar, each respondent received a foot care package. The local nurse conducted a weekly (between week-0 and week-4) visit of the respondents.

The third visit (week-4) was done for follow-up and problem solving. A 20-minute session of one to one discussion sought to identify any obstacle, personal feedback and positive support. A special meeting was done between the principal researcher, the local nurse and the high-risk respondents (as reported by the local nurse having little experience to perform the foot self-care behaviour effectively) with the help of their friend as a mentor (who was able to perform the effective behaviour) for skills improvements. Biweekly (between week-5 and week-12) the local nurse visited the respondents between the third and fourth visits.

At week-12, the respondents were evaluated for outcome measures by the research enumerator. The respondents were evaluated for the acceptability, compliance, effectiveness and maintenance towards this program. Continuous support and strong encouragement were given so they would perform the behaviour regularly. The local nurse was asked to share their experience regarding any limitations and to offer suggestions to improve the program.

For peer review only - http://bmjopen.bmj.com/site/about/guidelines.xhtml

Ethical procedures

This study has been approved by the Universiti Putra Malaysia Research Ethic Committee [Ref no. FPSK(FR15)P021] and the Social Welfare Department, Malaysia (Ref no. JKMM100/12/5/2:2015/003). This study was registered with the Australian New Zealand Clinical Trial Registry (ACTRN12616000210471) on 16 February 2016. The respondents were briefed on the study with a subject information sheet and consent taking done prior to data collection. The data was treated as private and confidential.

Statistical analysis

Data were analysed with descriptive and inferential statistics using Statistical Package for the Social Sciences (SPSS) version 20.0. Demographic data, clinical characteristics and acceptability profile were presented in mean and standard deviation (SD) or frequency (n) and percentage (%). Since the sample was small, non-parametric McNemar test and Wilcoxon signed-rank test were used to assess the outcomes.

For peer review only

For peer review only - http://bmjopen.bmj.com/site/about/guidelines.xhtml

RESULTS

Respondents' characteristics and feasibility of the study

There was a total of 52 older people with diabetes. One of them refused to participate for non-specific reasons. Therefore, 51 were screened for eligibility and 21 were excluded. The reasons for exclusion included; bedridden (5), cognitively impaired (6), known dementia (3), language barrier (2), depressed (1), known psychosis (2), deaf/mute (1), and blind (1). Hence, 31 respondents (60.8%) were eligible and agreed to participate (see Figure 1).

On average, the age of the respondents was 69 years (SD=4.23), the majority of them were female (54.8%), Malay (58.1%), attended primary school (51.6%), married (61.3%) and did not have children (61.3%). The average duration of the respondents living in the institution was five years (SD=3.84) (see Table 1).

Insert Table 1 here

On average, the respondents' FBG was 8.66 mmol/L (SD=3.15) and have been diagnosed with diabetes for 12 years (SD=12.95). Most of them were on oral medication(s) (74.2%), have comorbid disease(s) (93.5%) and never received any diabetes education (71.0%), all of them had no history of hospitalisation related to diabetes in the three months before data collection (100.0%), and 54.8% of them were non-smokers (see Table 2).

Insert Table 2 here

The recruitment rate and retention rate was 100% as all respondents were enrolled and completed the 12-week program. Figure 1 shows the flow diagram of enrolment, intervention, follow-up and analysis of the study. The figure was modified from the CONSORT 2010 Flow Diagram.[34]

Insert Figure 1 here

For peer review only

For peer review only - http://bmjopen.bmj.com/site/about/guidelines.xhtml

Acceptability of the study

On average, the acceptability score was moderately high (mean=33.84±4.08). The majority of the respondents reported that the program was acceptable (mean=4.32±0.48), effective (mean=4.06±0.81) and can be applied to other older diabetics (mean=4.29±0.46). The respondents liked this program, and considered the program a good way to prevent diabetic foot problems (mean=4.32±0.48), was helpful and had no adverse effects. However, the respondents were unsure whether they will continue to perform the behaviour after this program (mean=3.87±0.81) (see Table 3).

Insert Table 3 here

Foot self-care behaviour, foot care self-efficacy (efficacy-expectation), foot care outcome expectation, knowledge on foot care, quality of life and FBG

Table 4 shows the normality assessment of the data using the Shapiro-Wilks test of normality. The analysis indicated that, majority of the variable pairs, did not having a normal distribution data since the Shapiro-Wilks test was significant (i.e. p-value <0.05). Therefore, the nonparametric test (i.e. Wilcoxon Sign test) is a preferable test for assessing the significant difference between pre (i.e. baseline) and post (i.e. evaluation) variable score test.

Insert Table 4 here

Table 5 shows the foot self care behavior level were significantly increase from the baseline test (Median = 45.00) to evaluation test (Median = 69.00), Z = -4.86, p <0.001. Besides that, the Wilcoxon Sign test analysis also indicated that, the foot care self-efficacy (Median = 30.00), foot care outcome expectation (Median = 19.00), and also knowledge on foot score (Median = 8.00) at the baseline test were also statistically increase from the baseline test to evaluation test (foot care self-efficacy: Median = 44.00, Z = -4.76, p <0.001; foot care outcome expectation: Median = 25.00, Z = -4.79, p <0.001; knowledge on foot score: Median = 11.00, Z = -4.47, p <0.001).

For peer review only - http://bmjopen.bmj.com/site/about/guidelines.xhtml

The analysis reported in Table 5 also indicated that, at the baseline test, the score of quality of life for physical symptoms (Median = 23.00) and also FBG (Median = 7.90) were statistically decrease to evaluation test (Quality of life for physical symptoms: Median = 14.00, Z = -2.99, p =0.003; FBG: Median = 6.10, Z = -2.57, p =0.010). On the other hand, the analysis also indicated that, the score of quality of life for psychosocial functioning at the baseline test (Median = 15.00), do not statistically differ as compare to the score of evaluation test (Median = 26.00).

Insert Table 5 here

Foot condition

Table 6 shows that at baseline, most respondents had good foot hygiene (77.4%). The commonest foot condition was anhydrosis (61.3%) followed by skin fissures (29.0%), corns and callous (25.8%), nail infection (19.3%), skin injury (16.1%), involuted nail plates (12.9%) and dermatitis (6.5%). Only 3.2% of the respondents had ingrown toenails, subungual lesion, interdigital maceration, ulcers and amputated foot. Foot conditions improved significantly for overall foot hygiene (p=0.03) and anhydrosis (p=0.02) after the education program.

Insert Table 6 here

For peer review only

DISCUSSION

This study was conducted to determine the feasibility, acceptability and potential health-related impact of the foot self-care behaviour program. The rate of enrolment, attendance to the program and compliance are common indicators for assessing the feasibility of such a study.[31] The rate of enrolment was moderate (60.8%). The enrolment rate of this study was lower than other studies on education programs to enhance self-care practices among Malaysian adults with diabetes, which was ≥ 80%.[35] The differences could be due to the difference in inclusion criteria where our study focused on ab older population living in the long-term-care institution while other studies involved adults living in the community. Our results showed the rate of intervention attendance was high (100%), and no attrition among the respondents provides evidence that the program was feasible. This finding was similar to a theory-based pilot study on diabetes education conducted in Peninsular Malaysia among non-diabetic, aged ≥ 18 years and having at least secondary level of education.[36] They reported a response rate of 96.7% and all the respondents completed both pre- and post-test assessments.

The acceptability score was highly acceptable, and the finding was similar with other pilot studies conducted on foot self-care educational intervention among patients with diabetes in Canada.[31] The majority of the respondents in our study reported that the program was effective, beneficial, and enjoyable and perceived this program is a good way to prevent diabetic foot problems.

The study found there were improvements in the foot self-care behaviour, foot care self-efficacy (efficacy-expectation), foot care outcome expectation, knowledge on foot care and quality of life (physical symptoms) following the program. The foot self-care behaviour improved after 12 weeks following the education program. The findings were in line with previous interventional studies conducted on foot self-care among older diabetic population.[16 30 37-41]

For peer review only - http://bmjopen.bmj.com/site/about/guidelines.xhtml

The results showed that foot self-efficacy (efficacy-expectation) scores improved after implementing the education program. Previous studies demonstrated similar findings such as improvement in the foot self-efficacy scores between before and after implementation of foot self-care intervention program.[14-16] The respondents in our study reported being more confident in undertaking the foot self-care behaviour after the education program. Self-efficacy enhancing activities were integrated throughout the data collection process; starting at the seminar presentation, follow-up session and weekly reinforcement.

The foot care outcome expectation scores also improved from baseline to 12 weeks after the program. Likewise, the score for the outcome expectation improved after implementation of a self-efficacy intervention for patients with diabetes as found in other studies.[42] According to Bandura, outcome expectation is about a person's belief that when he performs a given behaviour, he will get the positive outcomes.[11]

The self-efficacy education program showed effectiveness in increasing knowledge on foot care at 12-week of the intervention. Similar to previous studies, it demonstrated that the knowledge level improved after the foot self-care education program. The diabetic foot self-care knowledge scores increased at one month,[16] three months,[38 43] six months,[41] and eight months[44] after intervention in previous studies. At baseline, the respondents in our study had moderate knowledge of risk factors for diabetic foot complications and foot self-care. Therefore, it can be suggested that older diabetics in the institution need further information regarding risks of diabetics' foot complication and the preventive measures.

There was a significant improvement in the respondents' quality of life on physical symptoms but not for the psychosocial functioning. This finding was similar to Aiken's study.[45] In contrast, Williams's stated that psychological well-being showed an improvement from baseline to 3 months post-intervention but not for physical health.[43] In other studies, the intervention did not lead to a significant effect on quality of life.[30 46] This is difficult to explain as the dimension of quality life is broad and concerns many aspects such as health status, socio-economic, culture, environment and spiritual.[47] The differences of quality of life in this study with other studies could be due to a different study location and population. As this study was conducted in a long-term public institution, other factors might influence the quality of life. For example, functional limitation to perform ADLs independently, disturbances in social relationship and disruption in emotional states among the residents and staff in the institution have an effect on psychosocial functioning.

For peer review only - http://bmjopen.bmj.com/site/about/guidelines.xhtml

The significant reduction in the FBG level in this study is encouraging, and this finding was similar to another Malaysian study that demonstrated the effects of self-management for diabetes patients by utilising the self-efficacy concept in their education program.[35]

The results showed improvements in overall foot hygiene and anhydrosis after the education program similar to Fan's study.[31] Other variables (i.e. nail conditions/ infections, corn or callous, skin fissure, skin injury and dermatitis) improved, but this change was not statistically significant. Likewise, there were no significant differences found in ulcer incidence and amputation at six months and 12 months post-intervention.[40] Fujiwara's study reported that callous grade improved at two-year post-intervention.[37] The differences in the outcome measure that influenced the effects of intervention could be due to the difference in the methodological approaches, sampling selection, instruments used, study duration or clinical practice.

Limitations of this study

There were several limitations of this study. It employed a non-randomised controlled trial (RCTs), and since there was no control group, there was a high risk of potential biases. The sample was relatively small, and a control group was not available to determine the effectiveness of study between groups. Lack of glycosylated haemoglobin (HbA1c) measurements may lead to insufficient information to determine the amount of respondents' blood glucose level for the previous three months. Self-reported in the outcome measurements may lead to recall and reporting bias. Other types of measurements such as data from medical record, biometric, or laboratory investigations would provide additional reliable and valid results. As this study was conducted among older diabetics living in the long-term-care institution, the findings are not able to be generalised to other populations.

For peer review only - http://bmjopen.bmj.com/site/about/guidelines.xhtml

CONCLUSION

These findings showed the program is feasible, acceptable and effective in improving older diabetics' foot self-care behaviour, foot care self-efficacy (efficacy-expectation), foot care outcome expectation, knowledge on foot care, quality of life in physical symptoms, FBG level and foot hygiene and anhydrosis after 12 weeks implementing the self-efficacy education program in a long-term institution in Selangor. Multidisciplinary collaboration between researchers, nurses and other health care providers in long-term institutions would help to determine patients' long-term goal of foot self-care behaviour.

Based on these findings, an education program based on self-efficacy theory would help and facilitate planning of a study in a larger older diabetes population living in long-term care institutions using RCT.

ACKNOWLEDGEMENTS

Special thanks to the Universiti Teknologi MARA and Ministry of Higher Education (MOHE) for PhD scholarship, to the Community Health Department, the Research Ethic Committee and the Putra Grant (no. 9463500). The authors would like to thank to Dr. Muhammad Mikhail Joseph Anthony Abdullah (Endocrinologist, Medical Department), Ms Nuraisyah Hani Zulkifley (Registered Nurse/ Masters student of Community Health Department) and Ms Nadia Alhana Arifin (Diabetic Nurse Educator, Family Medicine Department) Universiti Putra Malaysia, to the Social Welfare Department, Malaysia and to the nurses (Ms Nur Aidafitri Azahar, Ms Norfarahida Pami, Ms Norsyahida Ramli, Ms Sharifah Shahida Syed Azahar and Ms Azma Zain) involved in this study, the institution, and lastly the older diabetics who participated in the study.

CONTRIBUTORS Study design: SKAS, HAR, HSM, SSG; data collection: SKAS, HAR, SSG; data analysis: SKAS, MHAO, HAR, SSG and manuscript preparation: SKAS, HAR, HSM, SSG, MHAO.

CONFLICT OF INTERESTS

The authors declare no conflict of interest with regard to the research, authorship and publication of this study.

For peer review only - http://bmjopen.bmj.com/site/about/guidelines.xhtml

REFERENCES

1. International Diabetes Federation. IDF diabetes atlas sixth edition, 2013. www.idf.org/diabetesatlas
2. Leung PC. Diabetic foot ulcers - a comprehensive review. *The Royal Colleges of Surgeons of Edinburgh and Ireland* 2007;5:219-231.
3. Wild S, Roglic G, Green A, *et al.* Global prevalence of diabetes: estimates for the year 2000 and projections for 2030. *Diabetes Care* 2004;27:1047-1053.
4. National Health and Morbidity Survey 2011 (NHMS 2011). Ministry of Health Malaysia, 2011. http://www.moh.gov.my/index.php/pages/view/115
5. Feisul MI, Azmi S (Eds). National Diabetes Registry report, volume 1, 2009-2012. Kuala Lumpur; Ministry of Health Malaysia, 2013. http://www.moh.gov.my/index.php/pages/view/115
6. Mafauzy M, Hussein Z, Chan SP. The status of diabetes control in Malaysia : results of DiabCare 2008. *Medical Journal of Malaysia* 2011;66:175–181.
7. Visvanathan R, Zaiton A, Sherina MS, *et al.* The nutritional status of 1081 elderly people residing in publicly funded shelter homes in Peninsular Malaysia. *European Journal of Clinical Nutrition* 2005;59:318-324.
8. Dorresteijn J, Kriegsman D, Assendelft W, *et al.* Patient education for preventing diabetic foot ulceration. *The Cochrane Database of Systematic Reviews* 2010;5:CD001488.
9. Barlow J, Wright C, Sheasby J, *et al.* Self-management approaches for people with chronic conditions: a review. *Patient Education and Counseling* 2002;48:177-187.
10. Abdullah MAH, Abdullah AT. National Orthopaedic Registry Malaysia (NORM) 2009. 2010. http://www.acrm.org.my/norm
11. Bandura A. Self-efficacy: Toward a unifying theory of behavioral change. *Psychological Review* 1977;84:191–215.
12. Lenz ER, Shortridge-Baggett LM. *Self-efficacy in nursing: research and measurement perspectives.* New York: Springer Publishing Company. 2002.
13. Bandura A. *Social foundations of thought and action: a social cognitive theory.* New Jersey: Prentice Hall. 1986.
14. Seyyedrasooli A, Parvan K, Valizadeh L, *et al.* Self-efficacy in foot-care and effect of training: a single-blinded randomized controlled clinical trial. *International Journal of Community Based Nursing and Midwifery* 2015;3:141-149.

For peer review only - http://bmjopen.bmj.com/site/about/guidelines.xhtml

15. Fan L, Sidani S, Cooper-Brathwaite A, *et al.* Improving foot self-care knowledge, self-efficacy, and behaviors in patients with type 2 diabetes at low risk for foot ulceration: a pilot study. *Clinical Nursing Research* 2014;23:627-643

16. Borges W, Ostwald S. Behaviors with Pies Sanos. *Western Journal of Nursing Research* 2008;30:325-341.

17. van der Bijl JJ, Shortridge-Baggett LM. The theory and measurement of the self-efficacy construct. *Scholarly Inquiry for Nursing Practice* 2001;15:189-207.

18. Clinical Practice Guideline. Clinical Practice Guidelines 2015, management of type 2 diabetes mellitus (5th Edition). 2015. http://www.moh.gov.my/penerbitan/CPG/CPG%20T2DM%202015.pdf

19. International Working Group on the Diabetic Foot. International consensus on the diabetic foot. 2014. http://iwgdf.org/consensus/definitions-and-criteria/

20. O'Brien MJ, Perez A, Alos VA, *et al.* The feasibility, acceptability, and preliminary effectiveness of a Promotora-Led diabetes prevention program (PL-DPP) in Latinas: a pilot study. *The Diabetes Educator* 2015;41:485-494

21. Tarnowski KJ, Simonian SJ. Assessing treatment acceptance: The abbreviated Acceptability Rating Profile. *Journal Behaviour Theraphy and Experimental Psychiatry* 1992;23:101-106.

22. Chin YF, Huang TT, Hsu BRS. Impact of action cues, self-efficacy and perceived barriers on daily foot exam practice in type 2 diabetes mellitus patients with peripheral neuropathy. *Journal of Clinical Nursing* 2013;22:61-68.

23. Sloan HL. *Developing and testing the foot care confidence scale to measure self-efficacy in foot care. Dissertation Abstracts International: Section B: The Sciences and Engineering* 1998.

24. Wu SV, Courtney M, Edwards H, *et al.* Psychometric properties of the Chinese version of the perceived therapeutic efficacy scale for type 2 diabetes. *Journal Formos Medical Association* 2008;107:232-238.

25. Resnick B, Zimmerman SI, Orwig D, *et al.* Outcome expectations for exercise scale: utility and psychometrics. *Journal of Gerontology Social Sciences* 2000;55:S352-S356.

26. Desalu OO, Salawu FK, Jimoh AK, *et al.* Diabetic foot care: self-reported knowledge and practice among patients attending three tertiary hospital in Nigeria. *Ghana Medical Journal* 2011;45:60-65

27. Eigenmann C, Skinner T, Colagiuri R. Development and validation of a diabetes knowledge questionnaire. *Practical Diabetes International* 2011;*28:*166-170.

For peer review only - http://bmjopen.bmj.com/site/about/guidelines.xhtml

28. Pollock RD, Unwin NC, Connolly, V. Knowledge and practice of foot care in people with diabetes. *Diabetes Research and Clinical Practice* 2004;64:117-22.
29. Vileikyte L, Peyrot M, Bundy C. The Development and validation of a neuropathy- and foot ulcer – specific quality of life instrument. *Diabetes Care* 2003;26:2549-2555.
30. Baba M, Duff J, Foley L, *et al.* A comparison of two methods of foot health education: the Fremantle diabetes study phase II. *Primary Care Diabetes* 2015;9:155-162
31. Fan L, Sidani S, Cooper-Brathwaite A, *et al.* Feasibility, acceptability and effects of a foot self-care educational intervention on minor foot problems in adult patients with diabetes at low risk foot ulceration: a pilot study. *Canadian Journal of Diabetes* 2013;37:195-201
32. Lawshe CH. A quantitative approach to content validity. *Personnel psychology* 1975;28: 563-575
33. Johnston P, Wilkinson K. Enhancing validity of critical tasks selected for college and university program portfolios. *National Forum of Teacher Educational Journal* 2009;19:1-6.
34. The CONSORT 2010 Flow Diagram Transparent Reporting of Trial. 2010. http://www.consort-statement.org/consort-statement/flow-diagram
35. Tan MY, Magarey JM, Chee SS, *et al.* Brief structured education programme enhances self-care practices and improves glycaemic control in Malaysians with poorly controlled diabetes. *Health Education Research* 2011;26:896-907
36. Ahmad B, Ramadas A, Kia Fatt Q, *et al.* A pilot study: the development of a culturally tailored Malaysian Diabetes Education Module (MY-DEMO) based on the Health Belief Model. *BMC Endocrine Disorders* 2014;14:31.
37. Fujiwara Y, Kishida K, Terao M, *et al.* Beneficial effects of foot care nursing for people with diabetes mellitus: An uncontrolled before and after intervention study. *Journal of Advanced Nursing* 2011;67:1952-1962.
38. Saleh NA, Shebl A, Hatata E, *et al.* Impact of educational program about foot care on knowledge and self-care practice for diabetic older adult patients. *Journal of American Science* 2012;8:1444-1452.
39. Chen M, Huang W, Peng Y, *et al.* Effectiveness of a health promotion programme for farmers and fishermen with type-2 diabetes in Taiwan. *Journal of Advanced Nursing* 2011;67:2060-2067.
40. Lincoln N, Radford K, Game F, *et al.* Education for secondary prevention of foot ulcers in people with diabetes: a randomised controlled trial. *Diabetologia* 2008;51:1954-61.

For peer review only - http://bmjopen.bmj.com/site/about/guidelines.xhtml

41. Waxman R, Woodburn H, Powell M, *et al.* FOOTSTEP: a randomized controlled trial investigating the clinical and cost effectiveness of a patient self-management program for basic foot care in the elderly. *Journal of Clinical Epidemiology* 2003;56:1092-1099.

42. Wu SFV, Lee MC, Liang SY, *et al.* Effectiveness of a self-efficacy program for persons with diabetes: A randomized controlled trial. *Nursing and Health Sciences* 2011;13:335-343

43. Williams I, Utz S, Hinton I, *et al.* Enhancing diabetes self-care among rural African Americans with diabetes: results of a two-year culturally tailored intervention. *The Diabetes Educator* 2014;40:231-239.

44. Ko I, Lee T, Kim G, *et al.* Effects of visiting nurses' individually tailored education for low-income adult diabetic patients in Korea. *Public Health Nursing* 2011;28:429-437.

45. Aikens J, Rosland A, Piette J. Improvements in illness self-management and psychological distress associated with telemonitoring support for adults with diabetes. *Primary Care Diabetes* 2015;9:127-134.

46. Deakin T, Cade J, Williams R, *et al.* Structured patient education: The diabetes X-PERT programme makes a difference. *Diabetic Medicine* 2006;23:944–954.

47. Institute of Medicine (U.S.) Committee on improving quality in long-term care. improving the quality of long-term care. Washington, DC: National Academoies Press. 2001. http://nationalacademies.org/hmd/~/media/Files/Report%20Files/2003/Improving-the-Quality-of-Long-Term-Care/LTC8pagerFINAL.pdf

For peer review only

Title:

A Self-Efficacy Education Program on Foot Self-Care Behaviour among Older Diabetics in a Public Long-Term Care Institution, Malaysia: A Pilot Study

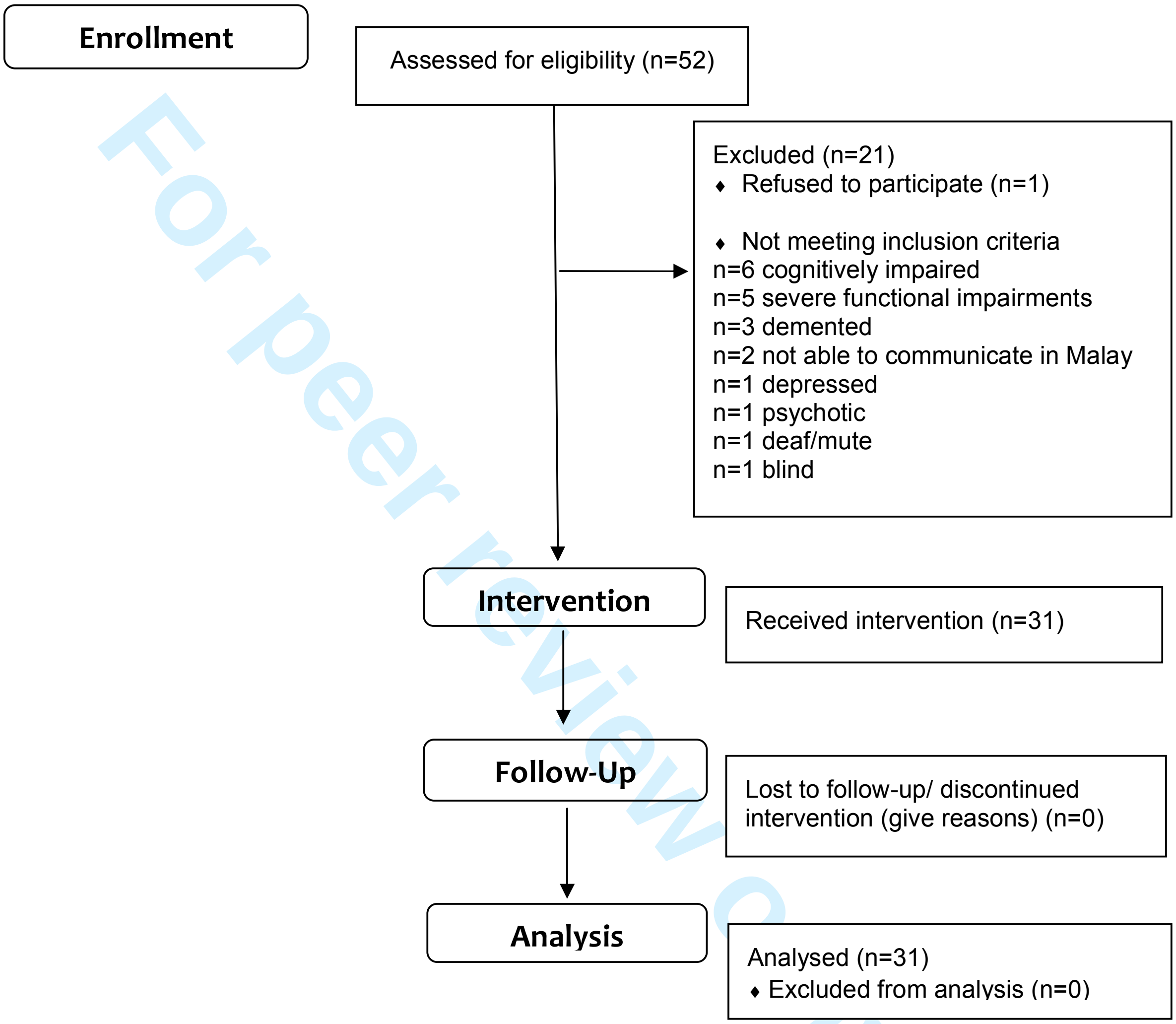

For peer review only

Figure 1 Flow Diagram of enrolment, intervention, follow up and analysis of the study.

The figure was modified from CONSORT 2010 Flow Diagram

Title:

A Self-Efficacy Education Program on Foot Self-Care Behaviour among Older Diabetics in a Public Long-Term Care Institution, Malaysia: A Pilot Study

Table 1

Distribution of respondents according to demographic characteristics (N=31)

Variables		n	%
Age	Mean±SD = 68.52 (4.23)		
Gender	Male	14	45.2
	Female	17	54.8
Ethnicity	Malay	18	58.1
	Chinese	3	9.7
	Indian	9	29.0
	Others	1	3.2
Education level	Never	4	12.9
	Primary	16	51.6
	Secondary	9	29
	Tertiary	2	6.5
Marital status	Single	12	38.7
	Married	19	61.3
Having children	No	19	61.3
	Yes	12	38.7
Duration of stay	Mean±SD = 4.84 (3.84)		

For peer review only

Table 2

Distribution of respondents according to clinical data (N=31)

Variables		n	%
FBG	Mean±SD = 8.66 (3.15)		
Diabetes duration	Mean±SD = 11.81 (12.95)		
Treatment	Oral	23	74.2
	Insulin	2	6.5
	Oral and insulin	6	19.4
Co-morbidity	No	2	6.5
	Yes	29	93.5
Had received previous diabetes education	No	22	71.0
	Foot care	1	3.2
	Others (e.g., diet, exercise, medication, blood glucose monitoring, smoking cessation)	8	25.8
Recent hospitalization (DM)	No	31	100.0
	Yes	0	0.0
Current smoking	No	17	54.8
	Yes	14	45.2

For peer review only

Table 3

The acceptability profile to the program delivered (N=31)

**Variables**	**Mean±SD**
This is an acceptable program for you	4.32±0.48
The program should be effective in changing the foot self-care behaviour	4.06±0.81
This program can be used for other older diabetics who did not performed foot self-care behaviour properly	4.29±0.46
You will continue to perform the foot self-care behaviour after this program	3.87±0.81
This program would not have bad side effects for you	4.32±0.48
You liked this program	4.32±0.48
The program was a good way to prevent diabetic foot problems	4.32±0.48
Overall, the program would help you	4.32±0.48
**Total score**	33.84±4.08

For peer review only

Table 4

Normality Assessment (N=31)

Variables	Shapiro-Wilks Test	
	Baseline	Week-12
Foot self-care behaviour	0.961	0.888*
Foot care self-efficacy (efficacy expectation)	0.986	0.927*
Foot care outcome expectation	0.955	0.872*
Knowledge on foot care	0.826*	0.759*
Quality of life		
Physical symptoms	0.834*	0.746*
Psychosocial functioning	0.737*	0.936
FBG	0.880*	0.922*

**P*<0.05

For peer review only

Table 5

Changes in the foot self-care behaviour, foot care self-efficacy (efficacy expectation), foot care outcome expectation, knowledge on foot care and quality of life before and FBG after the program (N=31)

Variables	Mean±SD (Median)		Wilcoxon signed-rank (Z)
	Baseline	Week-12	
Foot self-care behaviour	47.00±9.21 (45.00)	68.00±6.23 (69.00)	Z = -4.86, p=0.001*
Foot care self-efficacy (efficacy expectation)	29.90±8.68 (30.00)	43.68±4.94 (44.00)	Z = -4.76, p=0.001*
Foot care outcome expectation	19.58±4.26 (19.00)	25.97±3.43 (25.00)	Z = -4.79, p=0.001*
Knowledge on foot care	6.68±2.90 (8.00)	9.97±1.35 (11.00)	Z = -4.47, p=0.001*
Quality of life			
Physical symptoms	27.48±16.65 (23.00)	19.90±12.32 (14.00)	Z = -2.99, p=0.003*
Psychosocial functioning	30.84±25.75 (15.00)	27.58±6.72 (26.00)	Z = -0.31, p=0.754
FBG	8.66±3.15 (7.90)	6.98±2.18 (6.10)	Z= -2.57, p=0.010*

**P*<0.05

For peer review only

Table 6

Changes in the foot score before and after before and after the program (N=31)

Variables		n (%)		McNemar
		Baseline	Week-12	
Overall foot hygiene (clean, short nail)	Yes	24 (77.4)	28 (90.3)	0.03*
Nail conditions				
Nail infection	Yes	6 (19.3)	4 (12.9)	0.63
Ingrown toenail/s	Yes	1 (3.2)	0 (0.0)	1.00
Subungual lesion/s	Yes	1 (3.2)	0 (0.0)	1.00
Involuted of nail plate/s	Yes	4 (12.9)	4 (12.9)	1.00
Common skin conditions				
Corns and/ or callous	Yes	8 (25.8)	4 (12.9)	0.22
Skin fissures	Yes	9 (29.0)	5 (16.2)	0.34
Anhydrosis	Yes	19 (61.3)	10 (32.3)	0.02*
Skin injury/ cuts/ abrasions/ blisters	Yes	5 (16.1)	3 (9.7)	0.73
Other conditions and infections				
Dermatitis/ eczema/ psoriasis	Yes	2 (6.5)	1 (3.2)	0.50
Interdigital maceration	Yes	1 (3.2)	1 (3.2)	1.00
Complications				
Ulcer/s and amputated	Yes	1 (3.2)	1 (3.2)	1.00

*$P<0.05$

For peer review only

CONSORT 2010 checklist of information to include when reporting a randomised trial*

Section/Topic	Item No	Checklist item	Reported on page No
**Title and abstract**			
	1a	Identification as a randomised trial in the title	NA
	1b	Structured summary of trial design, methods, results, and conclusions (for specific guidance see CONSORT for abstracts)	1
**Introduction**			
Background and objectives	2a	Scientific background and explanation of rationale	3,4
	2b	Specific objectives or hypotheses	4
**Methods**			
Trial design	3a	Description of trial design (such as parallel, factorial) including allocation ratio	5
	3b	Important changes to methods after trial commencement (such as eligibility criteria), with reasons	5
Participants	4a	Eligibility criteria for participants	5
	4b	Settings and locations where the data were collected	5
Interventions	5	The interventions for each group with sufficient details to allow replication, including how and when they were actually administered	5,6,10
Outcomes	6a	Completely defined pre-specified primary and secondary outcome measures, including how and when they were assessed	6-8
	6b	Any changes to trial outcomes after the trial commenced, with reasons	-
Sample size	7a	How sample size was determined	-
	7b	When applicable, explanation of any interim analyses and stopping guidelines	NA
Randomisation:			
Sequence generation	8a	Method used to generate the random allocation sequence	NA
	8b	Type of randomisation; details of any restriction (such as blocking and block size)	NA
Allocation concealment mechanism	9	Mechanism used to implement the random allocation sequence (such as sequentially numbered containers), describing any steps taken to conceal the sequence until interventions were assigned	NA
Implementation	10	Who generated the random allocation sequence, who enrolled participants, and who assigned participants to interventions	NA
Blinding	11a	If done, who was blinded after assignment to interventions (for example, participants, care providers, those	NA

For peer review only - http://bmjopen.bmj.com/site/about/guidelines.xhtml

		assessing outcomes) and how	
	11b	If relevant, description of the similarity of interventions	NA
Statistical methods	12a	Statistical methods used to compare groups for primary and secondary outcomes	11
	12b	Methods for additional analyses, such as subgroup analyses and adjusted analyses	NA
**Results**			
Participant flow (a diagram is strongly recommended)	13a	For each group, the numbers of participants who were randomly assigned, received intended treatment, and were analysed for the primary outcome	12
	13b	For each group, losses and exclusions after randomisation, together with reasons	NA
Recruitment	14a	Dates defining the periods of recruitment and follow-up	12
	14b	Why the trial ended or was stopped	NA
Baseline data	15	A table showing baseline demographic and clinical characteristics for each group	12 (Table 1,2)
Numbers analysed	16	For each group, number of participants (denominator) included in each analysis and whether the analysis was by original assigned groups	-
Outcomes and estimation	17a	For each primary and secondary outcome, results for each group, and the estimated effect size and its precision (such as 95% confidence interval)	12, 13, 14
	17b	For binary outcomes, presentation of both absolute and relative effect sizes is recommended	-
Ancillary analyses	18	Results of any other analyses performed, including subgroup analyses and adjusted analyses, distinguishing pre-specified from exploratory	-
Harms	19	All important harms or unintended effects in each group (for specific guidance see CONSORT for harms)	NA
**Discussion**			
Limitations	20	Trial limitations, addressing sources of potential bias, imprecision, and, if relevant, multiplicity of analyses	17
Generalisability	21	Generalisability (external validity, applicability) of the trial findings	18
Interpretation	22	Interpretation consistent with results, balancing benefits and harms, and considering other relevant evidence	15, 16, 17
**Other information**			
Registration	23	Registration number and name of trial registry	1
Protocol	24	Where the full trial protocol can be accessed, if available	ACTRN12616000210471
Funding	25	Sources of funding and other support (such as supply of drugs), role of funders	2

*We strongly recommend reading this statement in conjunction with the CONSORT 2010 Explanation and Elaboration for important clarifications on all the items. If relevant, we also recommend reading CONSORT extensions for cluster randomised trials, non-inferiority and equivalence trials, non-pharmacological treatments, herbal interventions, and pragmatic trials. Additional extensions are forthcoming: for those and for up to date references relevant to this checklist, see www.consort-statement.org.

For peer review only

For peer review only - http://bmjopen.bmj.com/site/about/guidelines.xhtml

BMJ Open

A Self-Efficacy Education Program on Foot Self-Care Behaviour among Older Diabetics in a Public Long-Term Care Institution, Malaysia: A Quasi-Experimental Pilot Study

Journal:	*BMJ Open*
Manuscript ID	bmjopen-2016-014393.R1
Article Type:	Research
Date Submitted by the Author:	26-Jan-2017
Complete List of Authors:	Sharoni, Siti Khuzaimah Ahmad; Univ Teknol MARA, Nursing Department; University Putra Malaysia, Community Health Department<br>Abdul Rahman, Hejar; University Putra Malaysia, Community Health Department, Faculty of Medicine and Health Sciences<br>Minhat, Halimatus Sakdiah; University Putra Malaysia, Community Health Department, Faculty of Medicine and Health Sciences<br>Shariff Ghazali, Sazlina; University Putra Malaysia, Department of Family Medicine, Faculty of Medicine and Health Sciences<br>Azman Ong, Mohd Hanafi; Universiti Teknologi MARA, Department of Statistics, Faculty of Computer Science and Mathematics, UiTM Segamat Campus
**Primary Subject Heading**:	Diabetes and endocrinology
Secondary Subject Heading:	Geriatric medicine, Nursing, Public health
Keywords:	Diabetic foot < DIABETES & ENDOCRINOLOGY, PUBLIC HEALTH, GERIATRIC MEDICINE

SCHOLARONE™
Manuscripts

For peer review only

A Self-Efficacy Education Program on Foot Self-Care Behaviour among Older Diabetics in a Public Long-Term Care Institution, Malaysia: A Quasi-Experimental Pilot Study

International naming convention:

Sharoni, S.K.A, Rahman, H.A, Minhat, H. S., Ghazali, S.S, & Ong, M.H.A

Authors' addresses

Siti Khuzaimah Ahmad Sharoni[1,2], Hejar Abdul Rahman[2*], Halimatus Sakdiah Minhat[2], Sazlina Shariff Ghazali[3], Mohd Hanafi Azman Ong[4]

[1]Department of Nursing, Faculty of Health Sciences, Universiti Teknologi MARA, Puncak Alam Campus, 42300 Puncak Alam, Selangor, Malaysia

[2]Department of Community Health, Faculty of Medicine and Health Sciences, Universiti Putra Malaysia, 43400 UPM Serdang, Selangor, Malaysia

[3]Department of Family Medicine, Faculty of Medicine and Health Sciences, Universiti Putra Malaysia 43400 UPM Serdang, Selangor, Malaysia

[4]Department of Statistics, Faculty of Computer Science and Mathematics, Universiti Teknologi MARA, Segamat Campus, KM 12, Jalan Muar, 85000, Segamat, Johor, Malaysia.

Authors' information:

1) Siti Khuzaimah Ahmad Sharoni (MscN, RN)

Lecturer, Department of Nursing, Faculty of Health Sciences, Universiti Teknologi MARA, Puncak Alam Campus, 42300 Puncak Alam, Selangor, Malaysia

PhD student, Department of Community Health, Faculty of Medicine and Health Sciences, Universiti Putra Malaysia, 43400 UPM Serdang, Selangor, Malaysia

Email: sitik123@yahoo.com ; sitik123@salam.uitm.edu.my

Telephone: +603-3258 4349; Fax: +603-3258 4599

2) Associate Professor Dr Hejar Abdul Rahman (M. Com. Health) ***Corresponding author**

Associate Professor, Department of Community Health, Faculty of Medicine and Health Sciences, Universiti Putra Malaysia, 43400 UPM Serdang, Selangor, Malaysia

Email: hejar@upm.edu.my

Telephone: +603-8947 2413; Fax: +603-89450151

For peer review only - http://bmjopen.bmj.com/site/about/guidelines.xhtml

3) Associate Professor Dr Halimatus Sakdiah Minhat (DrPh)

Associate Professor, Department of Community Health, Faculty of Medicine and Health Sciences, Universiti Putra Malaysia, 43400 UPM Serdang, Selangor, Malaysia

Email: halimatus@upm.edu.my

Telephone: +603-8947 2417; Fax: +603-89450151

4) Associate Professor Dr Sazlina Shariff Ghazali (PhD, M. Med (Fam. Med),

Associate Professor, [3]Department of Family Medicine, Faculty of Medicine and Health Sciences, Universiti Putra Malaysia 43400 UPM Serdang, Selangor, Malaysia

Email: sazlina@upm.edu.my

Telephone: +603-8947 2532; Fax: +603-89472328

5) Mohd Hanafi Azman Ong (Msc. Applied Statistics)

Lecturer, Department of Statistics, Faculty of Computer Science and Mathematics, Universiti Teknologi MARA, Segamat Campus, KM 12, Jalan Muar, 85000, Segamat, Johor, Malaysia.

Email: napieong@yahoo.com

Telephone: +607-935 2000; Fax: +607-935 2277

Keywords: Diabetes, foot, long-term care, self-care behaviour, self-efficacy

Statistical summary of the manuscript:

Manuscript content: 4521 words

Abstract: 308 words

No of table/ figure: 6 tables, 1 Figure

No of references: 48 References

Data sharing statement: Our study protocol can be accessed freely available via https://www.anzctr.org.au/Trial/Registration/TrialReview.aspx?id=369488

For peer review only

ABSTRACT

Objective: A pilot self-efficacy education program was conducted to assess the feasibility, acceptability and potential impact of the self-efficacy education program on improving foot self-care behaviour among older diabetics in a public long-term care institution.

Method: A pre- and post- quasi-experimental study was conducted in a public long-term care institution in Selangor, Malaysia. Diabetes patients aged 60 years and above who fulfilled the selection criteria were invited to participate in this program. Four self-efficacy information sources; performance accomplishments, vicarious experience, verbal persuasion, and physiological information were translated into program interventions. The program consisted of four visits over a 12-week period. The first visit included screening and baseline assessment and the second visit involved 30 minutes of group seminar presentation. The third and fourth visits entailed a 20-minute one to one follow-up discussion and evaluation. A series of visits to the respondents was conducted throughout the program. The primary outcome was foot self-care behaviour. Foot self-efficacy (efficacy-expectation), foot care outcome expectation, knowledge of foot care, quality of life, fasting blood glucose and foot condition were secondary outcomes. Data were analysed with descriptive and inferential statistics (McNemar test and Wilcoxon signed-rank test) using the Statistical Package for the Social Sciences version 20.0.

Results: Fifty-two residents were recruited but only 31 met the inclusion criteria and were included in the analysis at baseline and at 12 weeks post-intervention. The acceptability rate was moderately high. At post-intervention, foot self-care behaviour ($p<0.001$), foot self-efficacy (efficacy-expectation), ($p<0.001$), foot care outcome expectation ($p<0.001$), knowledge of foot care ($p<0.001$), quality of life (physical symptoms) ($p=0.003$), fasting blood glucose ($p=0.010$), foot hygiene ($p=0.030$) and anhydrosis ($p=0.020$) showed significant improvements.

Conclusion: Findings from this pilot study would facilitate the planning of a larger study among older diabetic population living in long-term care institutions.

Trial registration number: ACTRN12616000210471

For peer review only

ARTICLE SUMMARY

Strengths and limitations of this study

- This is the first intervention program addressing foot self-care behaviour based on self-efficacy constructs among older diabetics living in a public long-term care institution in Malaysia.
- The self-efficacy education program was feasible, acceptable and successful in assisting older diabetics improve foot self-care behaviour and other health outcomes.
- The study highlights the need to conduct similar interventions among older diabetics living in long-term care institutions in Malaysia.
- The sample is relatively small, and the intervention employed a non-randomised and non-controlled trial, hence, the trial had high potential biases.
- Some of the outcome measurements were self-reported and may lead to recall and reporting biases. Other measurements such as data from medical records, biometrics, or laboratory investigations would provide additional reliable and valid results.

Funding statement:

This work was supported by Universiti Putra Malaysia (Puta Grant), grant number 9463500

For peer review only

For peer review only - http://bmjopen.bmj.com/site/about/guidelines.xhtml

INTRODUCTION

Diabetes is a common chronic disease affecting older people, and it is becoming a global health concern. The International Diabetes Federation reported that there are more than 134.6 million older diabetics worldwide and the number is expected to increase to 252.8 million by 2035.[1] The prevalence of diabetes is expected to increase exponentially in the next 20 years for developing countries.[2] By 2030, there would be more than 82 million older diabetics in developing countries.[3]

In Malaysia, the National Health Morbidity Survey reported that 15.2% (2.6 million) have beendiagnosed with diabetes.[4] The prevalence of diabetes among those aged 55 years and above was 45%.[5] Microvascular and severe late diabetic complications were reported at 75% and 25.4%, respectively.[6] The National Diabetes Registry reported that the prevalence of neuropathy, diabetic foot ulcer and amputation were at 70%, 11.1% and 11.0%, respectively. These diabetic complications have a significant impact especially for older populations.[5]

In Malaysia, the Ministry of Women, Family and Community Development abbreviated as KPWKM, manages public long-term care institutions. It was reported that 9% of the residents living in public long-term care institutions in Peninsular Malaysia have diabetes.[7] Older diabetics living in the institutions receive treatment from public health clinics under the Ministry of Health (MOH). However, little is known about diabetes management among older diabetics living in long-term care facilities.

Diabetes education is effective in improving foot self-care behaviour and preventing diabetic foot complications.[8] Self-care behaviour is the ability, knowledge, skills and confidence to make daily decisions.[9] Foot self-care behaviour is essential as this can improve health outcomes. However, foot self-care behaviour awareness among Malaysians with diabetes is relatively low. The National Orthopaedic Registry Malaysia reported that approximately 17.6% of diabetics attended diabetic foot clinics, 22.8% kept a diabetes booklet, 23.2% applied moisturising lotion on their feet, 26.5% wore appropriate footwear, and 27.3% received formal education on foot care.[10]

For peer review only

For peer review only - http://bmjopen.bmj.com/site/about/guidelines.xhtml

Self-efficacy is defined as confidence in one's ability to perform a particular behaviour, and is expected to influence the likelihood of the behavioural occurrence.[11, 12] The Self-efficacy theory refers to the individuals' belief, feelings and their motivation. The two essential components of this theory are the expectation of the individual's ability (self-efficacy or efficacy-expectation) and determination to practise specific behaviour (outcome expectations).[13] Previous intervention studies showed significant improvements in foot self-care behaviour after the implementation self-efficacy education strategies.[14-16] To date, few published intervention studies related to self-efficacy education programs focused on foot self-care behaviour among older diabetics living in a public long-term care institution in Malaysia.

AIM

The objective of this study was to assess the feasibility, acceptability and potential impact of the self-efficacy education program on improving foot self-care behaviour among older diabetics in a public long-term care institution. The primary outcome of the study was foot self-care behaviour. Foot care self-efficacy (efficacy-expectation), foot care outcome expectation, knowledge of foot care, quality of life, fasting blood glucose (FBG) and foot condition were the secondary outcomes.

For peer review only - http://bmjopen.bmj.com/site/about/guidelines.xhtml

METHOD

Design and sampling

This is a pre- and post-quasi-experimental study conducted in a public long-term care institution located in Selangor, Malaysia. During the period of data collection (January 2016), there was a total of 191 residents, of whom 52 had diabetes. Older diabetics aged ≥60 years, Malaysian, able to communicate in Malay and independent in the activities of daily living (ADLs) were invited to participate in this study. Older diabetics with cognitive impairment, psychosis, severe depression or blind, mute and deaf were excluded from this study. The principal researcher conducted a screening process prior to sample selection.

Elements of self-efficacy in the education program

This study incorporated self-efficacy constructs to develop the education program (Bandura, 1977). Self-efficacy in a person can be increased through four components: 1) performance accomplishments, 2) vicarious experience, 3) verbal persuasion, and 4) physiological information. Self-efficacy in diabetes management includes building new goals, starting with small steps, identifying specific needs, giving positive feedback and encouragement, and skills improvement and problem solving for respondents who are in difficult situations.[17]

In this study, self-efficacy constructs were integrated into the program through knowledge transfer (seminar presentations and pamphlets) and self-efficacy (self-confidence) enhancement activities. To enhance the respondents' self-efficacy, they were encouraged to develop their targets, work in small, realistic steps, be more focused, and have the confidence to perform the desired behaviour. A pamphlet (symbolic modelling) was provided for self-guidance. During follow-up meetings, the respondents who were reported as unable to perform the foot self-care behaviour received specific guidance and encouragement by the principal researcher to participate in the recommended lifestyle adjustments. A sharing session with the researcher and the local nurse was conducted among respondents who had difficulty in performing the behaviour effectively. Respondents who managed to perform the behaviour effectively were appointed as mentors (live modelling) to other respondents who were not able to do so. The respondents received weekly visits by the local nurse for physical and emotional support.

For peer review only - http://bmjopen.bmj.com/site/about/guidelines.xhtml

Intervention module

Knowledge transfer was conducted during the health education program. The respondents received information from PowerPoint presentations (PPT) (oral and visual information) and pamphlets (written and visual information). The program was conducted in the meeting room, consisted of 10-11 respondents/ group/ session, and was facilitated by the researcher. The education activities included a 20-minute PPT seminar aided by a pamphlet. The topic of this intervention program was "Foot self-care for older diabetics". The content and design of the PPT and pamphlet were adapted from standard diabetes organisations.[1,18,19] The content of the PPT and the pamphlet highlighted the risk factors of diabetes foot complications, foot self-examination, daily foot hygiene and cleanliness, foot protection and prevention of foot-related complications.

A foot care package consisting of a pamphlet on foot self-care, a nail clipper, a water-based lotion and a small towel was given to each respondent after the seminar. A reminder checklist was developed for the local nurse in charge of the clinic at the institution. The nurse also received instructions from the researcher to remind, give support and guidance to the respondents on performing the foot self-care behaviour daily. The respondents were advised to ask for guidance from the local nurse or their colleagues. The nurse was required to put her signature on the respondent's name column of the reminder checklist after visiting the respondents.

Variables and measurements

The feasibility of this study was measured by the respondents' recruitment, attendance, attrition and compliance rates.[20] The acceptability profile was evaluated after completing the 12-week program with a modified version of the Abbreviated Acceptability Rating Profile.[21] Eight items were used to assess respondents' acceptability of the self-efficacy education program. A 5-point Likert scale [strongly disagree (1), disagree (2), neither disagree or agree (3), agree (4) and strongly agree (5)] was used. The score ranged from 8 to 40 and a higher score indicated better acceptability towards the program delivered.

For peer review only - http://bmjopen.bmj.com/site/about/guidelines.xhtml

A questionnaire was used to evaluate the impact of the intervention on the outcome measures. The demographic data consisted of age, gender, ethnicity, education level, marital status, having children, and duration of stay in the institution. FBG, diabetes duration, treatment of diabetes, co-morbidity, previous diabetes education received, current smoking status and current history of hospitalisation related to diabetes were assessed for clinical characteristics.

The primary outcome of this study was foot self-care behaviour measured using the modified version of Diabetes Foot Self-Care Behaviour Scale (DFSBS).[22] The original DFSBS had good validity and reliability (Cronbach $\alpha = 0.73$), and the test-retest reliability was 0.92.[22] Foot self-care behaviour consisted of two parts. In part 1, seven items were asked about how many days the respondents performed foot self-care behaviour in the past seven days (one week). Part 2 (nine items) was about the frequency in which respondents performed a certain foot self-care behaviour. The responses were rated as a 5- point Likert scale [never/ 0 day per week (1), rarely/ 1-2days per week (2), sometimes/ 3-4 days per week (3), often/ 5-6 days per week (4) and always/ 7 days per week (5)].[22] The score ranged from 16 to 80; a higher score indicated good foot self-care behaviour.

For foot care self-efficacy (efficacy-expectation), the modified version of the Foot Care Confidence Scale (FCCS) was used.[23] The reliability test of the original FCCS was high (Cronbach $\alpha = 0.92$).[23] The foot care self-efficacy (efficacy-expectation), consisted of 10 items with a 5-point Likert scale [strongly not confident (1), not confident (2), moderate confident (3), confident (4) and strongly confident (5)]. The score ranged from 10 to 50; a higher score indicated higher confidence to perform the foot self-care behaviour.

Foot care outcome expectation was developed based on previous literature.[12 23 24 25] The scale consisted of six items with a 5-point Likert scale [strongly disagree (1), disagree (2), neither disagree or agree (3), agree (4) and strongly agree (5)]. The score ranged from 6 to 30 and a higher score indicated that the respondents had higher confidence in performing good foot self-care behaviour.

For peer review only - http://bmjopen.bmj.com/site/about/guidelines.xhtml

Knowledge of foot care was developed based on previous literature.[26-28] The instrument assessed the respondents' knowledge about risk factors, common diabetic foot complications and foot care. The scale consisted of 11 items with three possible answers (true, false and don't know). One mark was given for each correct answer. The score ranged from 0 to 11. A higher score indicated a higher level of knowledge.

The modified version of the Neuropathy and Foot Ulcer Specific Quality of Life (FS-QoL) was used to assess the quality of life of the respondents.[29] The original instrument showed a good reliability (Cronbach $\alpha = 0.86 - 0.95$). The instrument was divided into physical symptoms (13 items) and psychosocial functioning (12 items). This is an ordinal scale divided into two sections. First, the respondents need to respond to the foot problems affecting their well-being [always (3), sometimes (2), never (1)] and psychosocial functioning [every time (3), seldom (2), none (1) or agree (3), neither agree or disagree (2) and disagree (3)]. In the second section, the respondents were asked about whether the foot problems bother them [none (1), some (2), or very (3)]. The total score was calculated by multiplying the score in section one and section two. The score for physical symptoms ranged between 13 and 117 and for psychosocial functioning the score range was from 12 to 108. A lower score indicated a better quality of life.

Foot condition was developed from previous literature.[30 31] The instrument assessed overall hygiene, nail conditions, common skin conditions, other conditions and infections and complication. If respondents had a foot condition, it was rated as "1 point" for each component (can be either left or right).

For peer review only - http://bmjopen.bmj.com/site/about/guidelines.xhtml

Validity and reliability

The intervention module and the instruments were assessed for face and content validity. This was to ensure that the materials were appropriate for current local practice, cultural equivalent, and appropriate for the population and study location. Content Validity Ratio (CVR) of the instruments were assessed by six experts; one endocrinologist, two public health specialists, one family medicine specialist, one diabetic nurse educator and one older diabetic.

The formula CVR = (2ng / N) – 1, was used for validity conformity[32] where ng is the number of panel experts, who thinks that the item is good, and N is the total number of panel experts. In this approach, six panel experts were asked to indicate if a measurement item in a set of items is "essential" or "useful but not essential" or "not necessary" to the operationalisation of a theoretical construct.[33] "Essential" items or assessment tasks are ones that best represent the goal and are desired. The value lies between -1.00 to +1.00, where CVR = 0.00 means that 50% of the panel experts in a panel size of N believe that the portfolio task is essential and therefore valid (Johnston & Wilkinson, 2009).[33] In this study, the items were excluded when CVR < 0.00.

Forward and backward translations from English to Malay and back to English was performed by two bilingual translators certified by the Institute of Language and Literature Malaysia. The second draft of the questionnaire was weighted carefully before data collection commenced.

The results of internal consistency or reliability tests were acceptable for foot self-care behaviour (Cronbach $\alpha = 0.68$), foot care self-efficacy (efficacy-expectation) (Cronbach $\alpha = 0.91$), foot care outcome expectation (Cronbach $\alpha = 0.88$), knowledge of foot care (Cronbach $\alpha = 0.86$) and quality of life (physical symptoms, Cronbach $\alpha = 0.68$) (psychosocial functioning, Cronbach $\alpha = 0.68$).

Data collection procedures were conducted by the principal researcher (registered nurse), a local nurse (community nurse in charge of the clinic) and a research enumerator (registered nurse). The research enumerator received two hours of training with a manual file. The local nurse received a 30-minute briefing by the principal researcher regarding visiting procedures and checklist usage.

For peer review only - http://bmjopen.bmj.com/site/about/guidelines.xhtml

Data collection procedures

The data collection process consisted of four visits. During the first visit, the activities included a screening session of all older diabetics for inclusion and exclusion criteria, consent taking procedures and baseline assessments. The respondents received information about the study's objectives, procedures, benefits and potential harms from the main researcher. Data collection was conducted by a trained research enumerator from baseline to week-12.

The second visit was a 30-minute seminar presentation delivered by the principal researcher in a meeting room at the institution. The respondents were divided into three groups: group A (10 respondents), group B (10 respondents) and group C (11 respondents). The presentation was delivered three times (one group/ session). At the end of the seminar, each respondent received a foot care package. The local nurse conducted a weekly (between week-0 and week-4) visit to the respondents.

The third visit (week-4) was for follow-up and problem solving. A 20-minute session of one-to-one discussion sought to identify any obstacles and provided personal feedback and positive support. A special meeting was held between the principal researcher, the local nurse and respondents who had little experience in performing the foot self-care behaviour effectively (as reported by the local nurse). A respondent who could perform the effective behaviour was appointed as a mentor for skill improvement. Biweekly, (between week-5 and week-12) the local nurse visited the respondents between the third and fourth visits.

At week-12, the respondents were evaluated for outcome measures by the research enumerator. The respondents were evaluated for acceptability, compliance, effectiveness and maintenance towards this program. Continuous support and strong encouragement were given so they would perform the behaviour regularly. The local nurse was asked to share their experience regarding any limitations and to offer suggestions to improve the program.

For peer review only - http://bmjopen.bmj.com/site/about/guidelines.xhtml

Ethical procedures

This study has been approved by the Universiti Putra Malaysia Research Ethic Committee [Ref no. FPSK(FR15)P021] and the Social Welfare Department, Malaysia (Ref no. JKMM100/12/5/2:2015/003). This study was registered with the Australian New Zealand Clinical Trial Registry (ACTRN12616000210471) on the 16th of February 2016. The respondents were briefed on the study using the subject information sheet and consent were obtained prior to data collection. The data was treated as private and confidential.

Statistical analysis

Data were analysed with descriptive and inferential statistics using the Statistical Package for the Social Sciences (SPSS) version 20.0. Demographic data, clinical characteristics and acceptability profile were presented in mean and standard deviation (SD) or frequency (n) and percentage (%). Since the sample was small, non-parametric McNemar test and Wilcoxon signed-rank test were used to assess the outcomes.

For peer review only

RESULTS

Respondents' characteristics and feasibility of the study

There were 52 older residents with diabetes. One of them refused to participate due to a non-specific reason. Therefore, 51 were screened for eligibility and 21 were excluded. The reasons for exclusion included; bedridden (5), cognitively impaired (6), known dementia (3), language barrier (2), depressed (1), known psychosis (2), deaf/mute (1), and blind (1). Hence, 31 respondents (60.8%) were eligible and agreed to participate (see Figure 1).

On average, the age of respondents was 69 years (SD=4.23), most were female (54.8%), Malay (58.1%), attended primary school (51.6%), married (61.3%) and did not have children (61.3%). The average duration of the respondents living in the institution was five years (SD=3.84) (see Table 1).

Table 1

Distribution of respondents according to demographic characteristics (N=31)

Variables		n	%
Age	Mean±SD = 68.52 (4.23)		
Gender	Male	14	45.2
	Female	17	54.8
Ethnicity	Malay	18	58.1
	Chinese	3	9.7
	Indian	9	29.0
	Others	1	3.2
Education level	Never	4	12.9
	Primary	16	51.6
	Secondary	9	29
	Tertiary	2	6.5
Marital status	Single	12	38.7
	Married	19	61.3

For peer review only

Having children	No	19	61.3
	Yes	12	38.7
Duration of stay	Mean±SD = 4.84 (3.84)		

On average, the respondents' FBG was 8.66 mmol/L (SD=3.15) and have been diagnosed with diabetes for 12 years (SD=12.95). Most of them were on oral medication(s) (74.2%), have comorbid disease(s) (93.5%) and have never received any diabetes education (71.0%), all of them had no history of hospitalisation related to diabetes in the three months before data collection (100.0%), and 54.8% of them were non-smokers (see Table 2).

Table 2

Distribution of respondents according to clinical data (N=31)

Variables		n	%
FBG	Mean±SD = 8.66 (3.15)		
Diabetes duration	Mean±SD = 11.81 (12.95)		
Treatment	Oral	23	74.2
	Insulin	2	6.5
	Oral and insulin	6	19.4
Co-morbidity	No	2	6.5
	Yes	29	93.5
Had received previous diabetes education	No	22	71.0
	Foot care	1	3.2
	Others (e.g., diet, exercise, medication, blood glucose monitoring, smoking cessation)	8	25.8
Recent	No	31	100.0

For peer review only - http://bmjopen.bmj.com/site/about/guidelines.xhtml

hospitalization (DM)	Yes	0	0.0
Current smoking	No	17	54.8
	Yes	14	45.2

The recruitment rate and retention rate was 100% as all respondents were enrolled and completed the 12-week program. Figure 1 shows the flow diagram of enrolment, intervention, follow-up and analysis of the study. The figure was modified from the CONSORT 2010 Flow Diagram.[34]

Insert Figure 1 here

Acceptability of the study

On average, the acceptability score was moderately high (mean=33.84±4.08). A majority of the respondents reported that the program was acceptable (mean=4.32±0.48), effective (mean=4.06±0.81) and can be applied to other older diabetics (mean=4.29±0.46). The respondents liked this program, and considered the program a good way to prevent diabetic foot problems (mean=4.32±0.48), found it helpful and they had no adverse effects. However, the respondents were unsure if they will continue to perform the behaviour after this program (mean=3.87±0.81) (see Table 3).

Table 3

The acceptability profile to the program delivered (N=31)

Variables	Mean±SD
This is an acceptable program for you	4.32±0.48
The program should be effective in changing the foot self-care behaviour	4.06±0.81
This program can be used for other older diabetics who did not performed foot self-care behaviour properly	4.29±0.46
You will continue to perform the foot self-care behaviour after this program	3.87±0.81
This program would not have bad side effects for you	4.32±0.48
You liked this program	4.32±0.48
The program was a good way to prevent diabetic foot problems	4.32±0.48

For peer review only - http://bmjopen.bmj.com/site/about/guidelines.xhtml

Overall, the program would help you	4.32±0.48
**Total score**	33.84±4.08

Foot self-care behaviour, foot care self-efficacy (efficacy-expectation), foot care outcome expectation, knowledge of foot care, quality of life and FBG

The normality of the continuous data was assessed with Shapiro-Wilks test (Table 4) . The analysis indicated that a majority of the variables were normally distributed as the Shapiro-Wilks test was significant (p <0.05). Therefore, a nonparametric test (i.e. Wilcoxon Sign test) was the preferred test for assessing the significant difference between pre (i.e. baseline) and post (i.e. evaluation) variable score test.

Table 4

Normality Assessment (N=31)

Variables	Shapiro-Wilks Test	
	Baseline	Week-12
Foot self-care behaviour	0.961	0.888*
Foot care self-efficacy (efficacy expectation)	0.986	0.927*
Foot care outcome expectation	0.955	0.872*
Knowledge on foot care	0.826*	0.759*
Quality of life		
Physical symptoms	0.834*	0.746*
Psychosocial functioning	0.737*	0.936
FBG	0.880*	0.922*

*P<0.05

For peer review only - http://bmjopen.bmj.com/site/about/guidelines.xhtml

Table 5 shows that foot self-care behaviour levels significantly increased from the baseline assessment (Median = 45.00) to the evaluation test (Median = 69.00), Z = -4.86, p <0.001. Besides that, the Wilcoxon Sign test analysis also indicated that foot care self-efficacy (Median = 30.00), foot care outcome expectation (Median = 19.00), and knowledge of foot care scores (Median = 8.00) statistically increased from the baseline test to the evaluation test (foot care self-efficacy: Median = 44.00, Z = -4.76, p <0.001; foot care outcome expectation: Median = 25.00, Z = -4.79, p <0.001; knowledge of foot care score: Median = 11.00, Z = -4.47, p <0.001).

The analysis reported in Table 5 also indicated that compared to the baseline test, the score of quality of life for physical symptoms (Median = 23.00) and FBG (Median = 7.90) statistically decreased in the evaluation test (Quality of life for physical symptoms: Median = 14.00, Z = -2.99, p =0.003; FBG: Median = 6.10, Z = -2.57, p =0.010). Meanwhile, the score of quality of life for psychosocial functioning showed no significant difference between the baseline test (Median = 15.00) and the evaluation test (Median = 26.00).

Table 5

Changes in the foot self-care behaviour, foot care self-efficacy (efficacy expectation), foot care outcome expectation, knowledge on foot care and quality of life before and FBG after the program (N=31)

Variables	Mean±SD (Median)		Wilcoxon signed-rank (Z)
	Baseline	Week-12	
Foot self-care behaviour	47.00±9.21 (45.00)	68.00±6.23 (69.00)	Z = -4.86, p=0.001*
Foot care self-efficacy (efficacy expectation)	29.90±8.68 (30.00)	43.68±4.94 (44.00)	Z = -4.76, p=0.001*
Foot care outcome expectation	19.58±4.26 (19.00)	25.97±3.43 (25.00)	Z = -4.79, p=0.001*
Knowledge on foot care	6.68±2.90 (8.00)	9.97±1.35 (11.00)	Z = -4.47, p=0.001*

For peer review only - http://bmjopen.bmj.com/site/about/guidelines.xhtml

Quality of life			
Physical symptoms	27.48±16.65 (23.00)	19.90±12.32 (14.00)	Z = -2.99, p=0.003*
Psychosocial functioning	30.84±25.75 (15.00)	27.58±6.72 (26.00)	Z = -0.31, p=0.754
FBG	8.66±3.15 (7.90)	6.98±2.18 (6.10)	Z= -2.57, p=0.010*

**P*<0.05

Foot condition

Table 6 shows that at baseline, most respondents had good foot hygiene (77.4%). The most common foot condition was anhydrosis (61.3%) followed by skin fissures (29.0%), corns and callouses (25.8%), nail infection (19.3%), skin injury (16.1%), involuted nail plates (12.9%) and dermatitis (6.5%). Only 3.2% of the respondents had ingrown toenails, subungual lesion, interdigital maceration, ulcers and an amputated foot. Foot conditions improved significantly for overall foot hygiene (p=0.03) and anhydrosis (p=0.02) after the education program.

Table 6

Changes in the foot score before and after before and after the program (N=31)

Variables		n (%) Baseline	Week-12	McNemar
Overall foot hygiene (clean, short nail)	Yes	24 (77.4)	28 (90.3)	0.03*
Nail conditions				
Nail infection	Yes	6 (19.3)	4 (12.9)	0.63
Ingrown toenail/s	Yes	1 (3.2)	0 (0.0)	1.00
Subungual lesion/s	Yes	1 (3.2)	0 (0.0)	1.00

For peer review only - http://bmjopen.bmj.com/site/about/guidelines.xhtml

Involuted of nail plate/s	Yes	4 (12.9)	4 (12.9)	1.00
Common skin conditions				
Corns and/ or callous	Yes	8 (25.8)	4 (12.9)	0.22
Skin fissures	Yes	9 (29.0)	5 (16.2)	0.34
Anhydrosis	Yes	19 (61.3)	10 (32.3)	0.02*
Skin injury/ cuts/ abrasions/ blisters	Yes	5 (16.1)	3 (9.7)	0.73
Other conditions and infections				
Dermatitis/ eczema/ psoriasis	Yes	2 (6.5)	1 (3.2)	0.50
Interdigital maceration	Yes	1 (3.2)	1 (3.2)	1.00
Complications				
Ulcer/s and amputations of toes	Yes	1 (3.2)	1 (3.2)	1.00

*P<0.05

For peer review only

DISCUSSION

This study was conducted to determine the feasibility, acceptability and potential health-related impact of the foot self-care behaviour program. The rate of enrolment, attendance to the program and compliance are common indicators for assessing the feasibility of such a study.[31] The rate of enrolment was moderate (60.8%). The enrolment rate of this study was lower when compared to another study on an education program that enhances self-care practices among Malaysian adults with diabetes, which was ≥ 80%.[35] This disparity could be due to the difference in the inclusion criteria, where our study focused on an older population living in a long-term-care institution while the other study involved adults living in the community. Our results showed that the rate of intervention attendance was high (100%), with no attrition among respondents, providing evidence that the program was feasible. This finding was similar to a theory-based pilot study on diabetes education conducted in Peninsular Malaysia among non-diabetics, aged ≥ 18 years and having at least secondary level of education.[36] They reported a response rate of 96.7% and all the respondents completed both pre- and post-test assessments.

The acceptability score was highly acceptable, and the finding was similar to a pilot study conducted on foot self-care educational intervention among patients with diabetes in Canada.[31] The majority of the respondents in our study reported that the program was effective, beneficial, and enjoyable and perceived this program as a good way to prevent diabetic foot problems.

The study found improvements in the foot self-care behaviour, foot care self-efficacy (efficacy-expectation), foot care outcome expectation, knowledge of foot care and quality of life (physical symptoms) following the program. Foot self-care behaviour improved after 12 weeks following the education program. The findings were in line with previous interventional studies conducted on foot self-care among older diabetic population.[16 37-39]

For peer review only - http://bmjopen.bmj.com/site/about/guidelines.xhtml

The results showed that foot self-efficacy (efficacy-expectation) scores improved after implementing the education program. Previous studies demonstrated similar findings such as improvements in the foot self-efficacy scores before and after the implementation of foot self-care intervention program.[14 15] The respondents in our study reported of being more confident in undertaking foot self-care behaviour after the education program. Self-efficacy enhancing activities were integrated throughout the data collection process; starting from the seminar presentation, follow-up session and weekly reinforcement.

The foot care outcome expectation scores also improved from baseline to 12 weeks after the program. Likewise, the score for the outcome expectation improved after the implementation of a self-efficacy intervention for patients with diabetes, as found in another study.[40] According to Bandura, outcome expectation is about a person's belief in achieving positive outcomes when he/she performs a given behaviour.[11]

The self-efficacy education program showed effectiveness in increasing knowledge of foot care at week 12 of the intervention. Similar to previous studies, it demonstrated that knowledge level improved after the foot self-care education program. Diabetic foot self-care knowledge scores increased at three months,[15 37 41] six months,[37] and eight months[42] after intervention in previous studies. At baseline, the respondents in our study had a moderate knowledge of risk factors for diabetic foot complications and foot self-care. Therefore, it can be suggested that older diabetics in the institution need further information regarding the risks of diabetic foot complications and preventive measures.

There was a significant improvement in the respondents' quality of life for physical symptoms but not for psychosocial functioning. This finding was similar to Aiken's study.[43] In contrast, Williams's study stated that psychological well-being showed an improvement from baseline to 3 months post-intervention but not for physical health.[41] In another study, the intervention did not lead to a significant effect on the quality of life.[44] This is difficult to explain as the dimension of the quality of life is broad and concerns many aspects such as health status, socio-economic, culture, environment and spiritual.[45] The differences of quality of life in this study with other studies could be due to different study locations and populations. As this study was conducted in a long-term public institution, other factors might influence the quality of life. For example, functional limitation in performing ADLs independently, disturbances in social relationship and disruption in emotional states among the residents and staff in the institution could influence psychosocial functioning.

For peer review only - http://bmjopen.bmj.com/site/about/guidelines.xhtml

A significant reduction in the FBG level in this study is encouraging, and this finding was similar to another Malaysian study that demonstrated the effects of self-management for diabetes patients by utilising the self-efficacy concept in their education program.[35]

The results showed improvements in overall foot hygiene and anhydrosis after the education program, similar to Fan's study.[31] Other variables (i.e. nail conditions/ infections, corn or callous, skin fissure, skin injury and dermatitis) improved, but this change was not statistically significant. Likewise, there were no significant differences found in ulcer incidence and amputation at six months and 12 months post-intervention.[39] Fujiwara's study reported that callous grade improved at two-year post-intervention.[46] The differences in the outcome measures that influenced the effects of intervention could be due to the difference in the methodological approaches, sampling selection, instruments used, study duration or clinical practice.

The intervention module used in this program was designed from diabetes standard practice guidelines and was carefully weighted for older diabetics. The program activities, incorporated with the four self-efficacy components, were practical and acceptable for daily local practice. The pamphlet and foot care package provides the opportunity for them to carry out self-revision and individual practice. Seminar presentationsand the series of follow-ups with a reminder checklist and reinforcements demonstrated a significant effect in assisting the older diabetics perform foot self-care behaviour regularly. It was supported by recent clinical guidance and guidelines that diabetic foot problems can be prevented with foot care education, foot protection and therapeutic footwear as well as professional foot examination.[47 48]

The strength of this study is the older diabetics lived together in an institution and the regular visits by the local health care provider allowed discussions on the process of foot self-care behaviour modification. The respondents were able to share their experience of the program and support each other for sustainability. It is hoped that in the future, the findings of this pilot study could contribute to the implementation of a foot self-care behaviour education program based on the self-efficacy theory. Similar education programs involving more samples of older diabetics in public long-term care institutions are needed in the Malaysian context. Continuous support from the university and the Ministry of Women, Family and Community Development with the necessary resources to assist older diabetics can improve the health status.

For peer review only - http://bmjopen.bmj.com/site/about/guidelines.xhtml

Limitations of this study

There were several limitations in this study. It employed a non-randomised controlled trial (RCTs), and since there was no control group, there was a high risk of potential biases. The sample was relatively small, and a control group was not available to determine the effectiveness of study between groups. The lack of glycosylated haemoglobin (HbA1c) measurements may lead to insufficient information to determine the amount of respondents' blood glucose level for the previous three months. Self-reports on the outcome measurements may lead to recall and reporting biases. Other types of measurements such as data from medical records, biometrics, or laboratory investigations would provide additional reliable and valid results. As this study was conducted among older diabetics living in a long-term-care institution, the findings could not be generalised to other populations.

CONCLUSION

These findings showed that the program is feasible, acceptable and effective in improving older diabetics' foot self-care behaviour. Based on these findings, an education program based on the self-efficacy theory would help and facilitate the planning of a study in a larger older diabetes population living in long-term care institutions using RCT.

ACKNOWLEDGEMENTS

Special thanks to Universiti Teknologi MARA and the Ministry of Higher Education (MOHE) for the PhD scholarship, the Community Health Department, the Research Ethic Committee and the Putra Grant (no. 9463500). The authors would like to thank Dr. Muhammad Mikhail Joseph Anthony Abdullah (Endocrinologist, Medical Department), Ms Nuraisyah Hani Zulkifley (Registered Nurse/ Masters student of Community Health Department) and Ms Nadia Alhana Arifin (Diabetic Nurse Educator, Family Medicine Department) Universiti Putra Malaysia, the Social Welfare Department, Malaysia and the nurses (Ms Nur Aidafitri Azahar, Ms Norfarahida Pami, Ms Norsyahida Ramli, Ms Sharifah Shahida Syed Azahar and Ms Azma Zain) involved in this study, the institution, and lastly the older diabetics who participated in the study.

For peer review only

For peer review only - http://bmjopen.bmj.com/site/about/guidelines.xhtml

CONTRIBUTORS Study design: SKAS, HAR, HSM, SSG; data collection: SKAS, HAR, SSG; data analysis: SKAS, MHAO, HAR, SSG, HSM, and manuscript preparation: SKAS, HAR, SSG, HSM, MHAO.

CONFLICT OF INTEREST

The authors declare no conflict of interest with regards to the research, authorship and publication of this study.

For peer review only

REFERENCES

1. International Diabetes Federation. IDF diabetes atlas sixth edition, 2013. www.idf.org/diabetesatlas
2. Leung PC. Diabetic foot ulcers - a comprehensive review. *The Royal Colleges of Surgeons of Edinburgh and Ireland* 2007;5:219-231.
3. Wild S, Roglic G, Green A, *et al.* Global prevalence of diabetes: estimates for the year 2000 and projections for 2030. *Diabetes Care* 2004;27:1047-1053.
4. National Health and Morbidity Survey 2011 (NHMS 2011). Ministry of Health Malaysia, 2011. http://www.moh.gov.my/index.php/pages/view/115
5. Feisul MI, Azmi S (Eds). National Diabetes Registry report, volume 1, 2009-2012. Kuala Lumpur; Ministry of Health Malaysia, 2013. http://www.moh.gov.my/index.php/pages/view/115
6. Mafauzy M, Hussein Z, Chan SP. The status of diabetes control in Malaysia : results of DiabCare 2008. *Medical Journal of Malaysia* 2011;66:175–181.
7. Visvanathan R, Zaiton A, Sherina MS, *et al.* The nutritional status of 1081 elderly people residing in publicly funded shelter homes in Peninsular Malaysia. *European Journal of Clinical Nutrition* 2005;59:318-324.
8. Dorresteijn J, Kriegsman D, Assendelft W, *et al.* Patient education for preventing diabetic foot ulceration. *The Cochrane Database of Systematic Reviews* 2010;5:CD001488.
9. Barlow J, Wright C, Sheasby J, *et al.* Self-management approaches for people with chronic conditions: a review. *Patient Education and Counseling* 2002;48:177-187.
10. Abdullah MAH, Abdullah AT. National Orthopaedic Registry Malaysia (NORM) 2009. 2010. http://www.acrm.org.my/norm

11. Bandura A. Self-efficacy: Toward a unifying theory of behavioral change. *Psychological Review* 1977;84:191–215.
12. Lenz ER, Shortridge-Baggett LM. *Self-efficacy in nursing: research and measurement perspectives.* New York: Springer Publishing Company. 2002.
13. Bandura A. *Social foundations of thought and action: a social cognitive theory.* New Jersey: Prentice Hall. 1986.
14. Seyyedrasooli A, Parvan K, Valizadeh L, *et al.* Self-efficacy in foot-care and effect of training: a single-blinded randomized controlled clinical trial. *International Journal of Community Based Nursing and Midwifery* 2015;3:141-149.
15. Fan L, Sidani S, Cooper-Brathwaite A, *et al.* Improving foot self-care knowledge, self-efficacy, and behaviors in patients with type 2 diabetes at low risk for foot ulceration: a pilot study. *Clinical Nursing Research* 2014;23:627-643
16. Borges W, Ostwald S. Behaviors with Pies Sanos. *Western Journal of Nursing Research* 2008;30:325-341.
17. van der Bijl JJ, Shortridge-Baggett LM. The theory and measurement of the self-efficacy construct. *Scholarly Inquiry for Nursing Practice* 2001;15:189-207.
18. Clinical Practice Guideline. Clinical Practice Guidelines 2015, management of type 2 diabetes mellitus (5th Edition). 2015. http://www.moh.gov.my/penerbitan/CPG/CPG%20T2DM%202015.pdf
19. International Working Group on the Diabetic Foot. International consensus on the diabetic foot. 2014. http://iwgdf.org/consensus/definitions-and-criteria/
20. O'Brien MJ, Perez A, Alos VA, *et al.* The feasibility, acceptability, and preliminary effectiveness of a Promotora-Led diabetes prevention program (PL-DPP) in Latinas: a pilot study. *The Diabetes Educator* 2015;41:485-494
21. Tarnowski KJ, Simonian SJ. Assessing treatment acceptance: The abbreviated Acceptability Rating Profile. *Journal Behaviour Theraphy and Experimental Psychiatry* 1992;23:101-106.
22. Chin YF, Huang TT, Hsu BRS. Impact of action cues, self-efficacy and perceived barriers on daily foot exam practice in type 2 diabetes mellitus patients with peripheral neuropathy. *Journal of Clinical Nursing* 2013;22:61-68.
23. Sloan HL. *Developing and testing the foot care confidence scale to measure self-efficacy in foot care. Dissertation Abstracts International: Section B: The Sciences and Engineering* 1998.

For peer review only

For peer review only - http://bmjopen.bmj.com/site/about/guidelines.xhtml

24. Wu SV, Courtney M, Edwards H, *et al.* Psychometric properties of the Chinese version of the perceived therapeutic efficacy scale for type 2 diabetes. *Journal Formos Medical Association* 2008;107:232-238.

25. Resnick B, Zimmerman SI, Orwig D, *et al.* Outcome expectations for exercise scale: utility and psychometrics. *Journal of Gerontology Social Sciences* 2000;55:S352-S356.

26. Desalu OO, Salawu FK, Jimoh AK, *et al.* Diabetic foot care: self-reported knowledge and practice among patients attending three tertiary hospital in Nigeria. *Ghana Medical Journal* 2011;45:60-65

27. Eigenmann C, Skinner T, Colagiuri R. Development and validation of a diabetes knowledge questionnaire. *Practical Diabetes International* 2011;*28:*166-170.

28. Pollock RD, Unwin NC, Connolly, V. Knowledge and practice of foot care in people with diabetes. *Diabetes Research and Clinical Practice* 2004;64:117-22.

29. Vileikyte L, Peyrot M, Bundy C. The Development and validation of a neuropathy- and foot ulcer – specific quality of life instrument. *Diabetes Care* 2003;26:2549-2555.

30. Baba M, Duff J, Foley L, *et al.* A comparison of two methods of foot health education: the Fremantle diabetes study phase II. *Primary Care Diabetes* 2015;9:155-162

31. Fan L, Sidani S, Cooper-Brathwaite A, *et al.* Feasibility, acceptability and effects of a foot self-care educational intervention on minor foot problems in adult patients with diabetes at low risk foot ulceration: a pilot study. *Canadian Journal of Diabetes* 2013;37:195-201

32. Lawshe CH. A quantitative approach to content validity. *Personnel psychology* 1975;28: 563-575

33. Johnston P, Wilkinson K. Enhancing validity of critical tasks selected for college and university program portfolios. *National Forum of Teacher Educational Journal* 2009;19:1-6.

34. The CONSORT 2010 Flow Diagram Transparent Reporting of Trial. 2010. http://www.consort-statement.org/consort-statement/flow-diagram

35. Tan MY, Magarey JM, Chee SS, *et al.* Brief structured education programme enhances self-care practices and improves glycaemic control in Malaysians with poorly controlled diabetes. *Health Education Research* 2011;26:896-907

36. Ahmad B, Ramadas A, Kia Fatt Q, *et al.* A pilot study: the development of a culturally tailored Malaysian Diabetes Education Module (MY-DEMO) based on the Health Belief Model. *BMC Endocrine Disorders* 2014;14:31.

For peer review only

37. Saleh NA, Shebl A, Hatata E, *et al.* Impact of educational program about foot care on knowledge and self-care practice for diabetic older adult patients. *Journal of American Science* 2012;8:1444-1452.

38. Chen M, Huang W, Peng Y, *et al.* Effectiveness of a health promotion programme for farmers and fishermen with type-2 diabetes in Taiwan. *Journal of Advanced Nursing* 2011;67:2060-2067.

39. Lincoln N, Radford K, Game F, *et al.* Education for secondary prevention of foot ulcers in people with diabetes: a randomised controlled trial. *Diabetologia* 2008;51:1954-61.

40. Wu SFV, Lee MC, Liang SY, *et al.* Effectiveness of a self-efficacy program for persons with diabetes: A randomized controlled trial. *Nursing and Health Sciences* 2011;13:335-343

41. Williams I, Utz S, Hinton I, *et al.* Enhancing diabetes self-care among rural African Americans with diabetes: results of a two-year culturally tailored intervention. *The Diabetes Educator* 2014;40:231-239.

42. Ko I, Lee T, Kim G, *et al.* Effects of visiting nurses' individually tailored education for low-income adult diabetic patients in Korea. *Public Health Nursing* 2011;28:429-437.

43. Aikens J, Rosland A, Piette J. Improvements in illness self-management and psychological distress associated with telemonitoring support for adults with diabetes. *Primary Care Diabetes* 2015;9:127-134.

44. Deakin T, Cade J, Williams R, *et al.* Structured patient education: The diabetes X-PERT programme makes a difference. *Diabetic Medicine* 2006;23:944–954.

45. Institute of Medicine (U.S.) Committee on improving quality in long-term care. improving the quality of long-term care. Washington, DC: National Academoies Press. 2001. http://nationalacademies.org/hmd/~/media/Files/Report%20Files/2003/Improving-the-Quality-of-Long-Term-Care/LTC8pagerFINAL.pdf

46. Fujiwara Y, Kishida K, Terao M, *et al.* Beneficial effects of foot care nursing for people with diabetes mellitus: An uncontrolled before and after intervention study. *Journal of Advanced Nursing* 2011;67:1952-1962.

47. Hingorani A, LaMuraglia GM, Henke P, *et al.* The management of diabetic foot: A clinical practice guideline by the Society for Vascular Surgery in collaboration with the American Podiatric Medical Association and the Society for Vascular Medicine, *Journal of Vascular Surgery* 2016; 63: 3S–21S

48. Bus SA, Van Netten JJ, Lavery LA, *et al.* IWGDF guidance on the prevention of foot ulcers in at-risk patients with diabetes. *Diabetes Metab Res Rev* 2015.

For peer review only - http://bmjopen.bmj.com/site/about/guidelines.xhtml

For peer review only

For peer review only - http://bmjopen.bmj.com/site/about/guidelines.xhtml

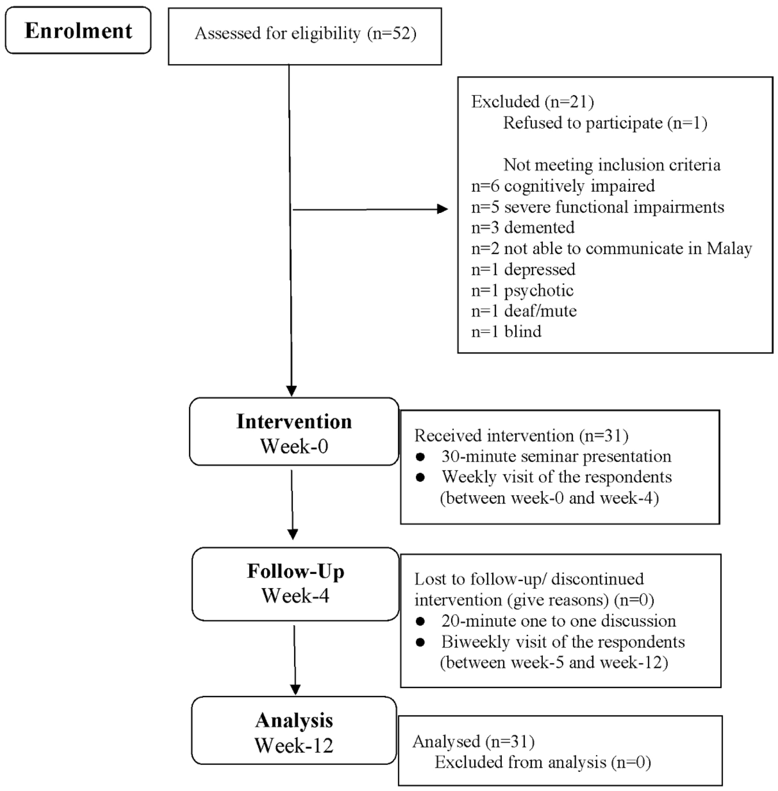

Figure 1 Flow Diagram of enrolment, intervention, follow up and analysis of the study.

The figure was modified from CONSORT 2010 Flow Diagram

172x233mm (300 x 300 DPI)

STROBE Statement—Checklist of items that should be included in reports of ***cohort studies***

	Item No	Recommendation	Reported on page No
**Title and abstract**	1	(*a*) Indicate the study's design with a commonly used term in the title or the abstract	1, 3
		(*b*) Provide in the abstract an informative and balanced summary of what was done and what was found	1, 3
**Introduction**			
Background /rationale	2	Explain the scientific background and rationale for the investigation being reported	5, 6
Objectives	3	State specific objectives, including any prespecified hypotheses	6
**Methods**			
Study design	4	Present key elements of study design early in the paper	7
Setting	5	Describe the setting, locations, and relevant dates, including periods of recruitment, exposure, follow-up, and data collection	7,12
Participants	6	(*a*) Give the eligibility criteria, and the sources and methods of selection of participants. Describe methods of follow-up	7, 12
		(*b*) For matched studies, give matching criteria and number of exposed and unexposed	-
Variables	7	Clearly define all outcomes, exposures, predictors, potential confounders, and effect modifiers. Give diagnostic criteria, if applicable	8-10
Data sources/ measurement	8*	For each variable of interest, give sources of data and details of methods of assessment (measurement). Describe comparability of assessment methods if there is more than one group	-
Bias	9	Describe any efforts to address potential sources of bias	-
Study size	10	Explain how the study size was arrived at	-
Quantitative variables	11	Explain how quantitative variables were handled in the analyses. If applicable, describe which groupings were chosen and why	13
Statistical methods	12	(*a*) Describe all statistical methods, including those used to control for confounding	13
		(*b*) Describe any methods used to examine subgroups and interactions	-
		(*c*) Explain how missing data were addressed	-
		(*d*) If applicable, explain how loss to follow-up was addressed	-
		(*e*) Describe any sensitivity analyses	-
**Results**			
Participants	13*	(a) Report numbers of individuals at each stage of study—eg numbers potentially eligible, examined for eligibility, confirmed eligible, included in the study, completing follow-up, and analysed	14
		(b) Give reasons for non-participation at each stage	14
		(c) Consider use of a flow diagram	Figure 1
Descriptive data	14*	(a) Give characteristics of study participants (eg demographic, clinical, social) and information on exposures and potential confounders	14
		(b) Indicate number of participants with missing data for each variable of interest	-
		(c) Summarise follow-up time (eg, average and total amount)	15,16
Outcome data	15*	Report numbers of outcome events or summary measures over time	15,16

For peer review only - http://bmjopen.bmj.com/site/about/guidelines.xhtml

data			
Main results	16	(*a*) Give unadjusted estimates and, if applicable, confounder-adjusted estimates and their precision (eg, 95% confidence interval). Make clear which confounders were adjusted for and why they were included	15,16
		(*b*) Report category boundaries when continuous variables were categorized	15,16
		(*c*) If relevant, consider translating estimates of relative risk into absolute risk for a meaningful time period	-
Other analyses	17	Report other analyses done—eg analyses of subgroups and interactions, and sensitivity analyses	-
**Discussion**			
Key results	18	Summarise key results with reference to study objectives	17-19
Limitations	19	Discuss limitations of the study, taking into account sources of potential bias or imprecision. Discuss both direction and magnitude of any potential bias	20
Interpretation	20	Give a cautious overall interpretation of results considering objectives, limitations, multiplicity of analyses, results from similar studies, and other relevant evidence	17-19
Generalisability	21	Discuss the generalisability (external validity) of the study results	-
**Other information**			
Funding	22	Give the source of funding and the role of the funders for the present study and, if applicable, for the original study on which the present article is based	20

*Give information separately for exposed and unexposed groups.

Note: An Explanation and Elaboration article discusses each checklist item and gives methodological background and published examples of transparent reporting. The STROBE checklist is best used in conjunction with this article (freely available on the Web sites of PLoS Medicine at http://www.plosmedicine.org/, Annals of Internal Medicine at http://www.annals.org/, and Epidemiology at http://www.epidem.com/). Information on the STROBE Initiative is available at http://www.strobe-statement.org.

For peer review only
